# Key aspects of papillomavirus infection influence the host cervicovaginal microbiome in a preclinical murine papillomavirus (MmuPV1) infection model

**DOI:** 10.1128/mbio.00933-24

**Published:** 2024-05-14

**Authors:** Megan E. Spurgeon, Elizabeth C. Townsend, Simon Blaine-Sauer, Stephanie M. McGregor, Mark Horswill, Johan A. den Boon, Paul Ahlquist, Lindsay Kalan, Paul F. Lambert

**Affiliations:** 1 McArdle Laboratory for Cancer Research, School of Medicine and Public Health, University of Wisconsin-Madison, Madison, Wisconsin, USA; 2 Department of Medical Microbiology and Immunology, School of Medicine and Public Health, University of Wisconsin-Madison, Madison, Wisconsin, USA; 3 Medical Scientist Training Program, School of Medicine and Public Health, University of Wisconsin-Madison, Madison, Wisconsin, USA; 4 Microbiology Doctoral Training Program, University of Wisconsin-Madison, Madison, Wisconsin, USA; 5 Department of Pathology and Laboratory Medicine, School of Medicine and Public Health, University of Wisconsin-Madison, Madison, Wisconsin, USA; 6 John W. and Jeanne M. Rowe Center for Research in Virology, Morgridge Institute for Research, Madison, Wisconsin, USA; 7 Department of Biochemistry and Biomedical Sciences, McMaster University, Hamilton, Ontario, Canada; 8 M.G. DeGroote Institute for Infectious Disease Research, McMaster University, Hamilton, Ontario, Canada; 9 David Braley Centre for Antibiotic Discovery, McMaster University, Hamilton, Ontario, Canada; The University of North Carolina at Chapel Hill, Chapel Hill, North Carolina, USA

**Keywords:** cervix, vagina, mouse papillomavirus, Mus musculus, papillomavirus, microbiome

## Abstract

Human papillomaviruses (HPVs) are the most common sexually transmitted infection in the United States and are a major etiological agent of cancers in the anogenital tract and oral cavity. Growing evidence suggests changes in the host microbiome are associated with the natural history and ultimate outcome of HPV infection. We sought to define changes in the host cervicovaginal microbiome during papillomavirus infection, persistence, and pathogenesis using the murine papillomavirus (MmuPV1) cervicovaginal infection model. Cervicovaginal lavages were performed over a time course of MmuPV1 infection in immunocompetent female *FVB/N* mice and extracted DNA was analyzed by qPCR to track MmuPV1 viral copy number. 16S ribosomal RNA (rRNA) gene sequencing was used to determine the composition and diversity of microbial communities throughout this time course. We also sought to determine whether specific microbial communities exist across the spectrum of MmuPV1-induced neoplastic disease. We, therefore, performed laser-capture microdissection to isolate regions of disease representing all stages of neoplastic disease progression (normal, low- and high-grade dysplasia, and cancer) from female reproductive tract tissue sections from MmuPV1-infected mice and performed 16S rRNA sequencing. Consistent with other studies, we found that the natural murine cervicovaginal microbiome is highly variable across different experiments. Despite these differences in initial microbiome composition between experiments, we observed that MmuPV1 persistence, viral load, and severity of disease influenced the composition of the cervicovaginal microbiome. These studies demonstrate that papillomavirus infection can alter the cervicovaginal microbiome.

**IMPORTANCE:**

Human papillomaviruses (HPVs) are the most common sexually transmitted infection in the United States. A subset of HPVs that infect the anogenital tract (cervix, vagina, anus) and oral cavity cause at least 5% of cancers worldwide. Recent evidence indicates that the community of microbial organisms present in the human cervix and vagina, known as the cervicovaginal microbiome, plays a role in HPV-induced cervical cancer. However, the mechanisms underlying this interplay are not well-defined. In this study, we infected the female reproductive tract of mice with a murine papillomavirus (MmuPV1) and found that key aspects of papillomavirus infection and disease influence the host cervicovaginal microbiome. This is the first study to define changes in the host microbiome associated with MmuPV1 infection in a preclinical animal model of HPV-induced cervical cancer. These results pave the way for using MmuPV1 infection models to further investigate the interactions between papillomaviruses and the host microbiome.

## INTRODUCTION

Human papillomaviruses (HPVs) are the most common sexually transmitted infection in the United States and are a significant and costly public health burden (1, 2). It is estimated that most sexually active individuals are exposed to sexually transmitted HPVs during their lifetime with 13 million new infections each year in the United States alone (2). A subset of oncogenic, high-risk HPVs cause approximately 5% of all human cancers, including nearly all cervical cancers, other anogenital cancers (e.g., vaginal, penile, and anal cancers), and a growing fraction of head and neck cancers (3, 4). Despite the availability of highly effective prophylactic HPV vaccines, cervical cancer continues to be a leading cause of death by cancer in women worldwide (5–7). Persistent HPV infections, particularly with high viral loads, are highly predictive for cervical carcinogenesis and neoplastic progression (8–12). However, HPV infection alone is not sufficient for carcinogenesis. Therefore, there is a need to identify factors that contribute to infection, persistence, and malignant progression caused by these common human pathogens.

One such factor may be the microbiome. In women, a healthy cervicovaginal microbiome (CVM) is often defined as having low bacterial diversity with a predominance of one or more *Lactobacillus* species (13–15), which help prevent pathogen colonization in a variety of ways (16). Several studies have shown that deviation from a healthy, *Lactobacillus*-dominant CVM correlates with HPV infection, persistence, and neoplastic development in the female reproductive tract (17–25). Although there is variability across studies, in general, HPV infection and disease are often associated with more highly diverse and *Lactobacillus*-depleted CVMs colonized with anaerobic bacterial taxa (e.g., *Gardnerella vaginalis* and species of *Prevotella*, *Sneathia,* and *Megasphaera*) (17, 21–23, 26–28). These anaerobic bacterial species are also present in bacterial vaginosis (BV), a highly prevalent vaginal dysbiosis observed in reproductive-aged women. Hallmarks of BV are excessive polymicrobial biofilm production and vaginal discharge, elevated pH, reproductive complications, and increased susceptibility to sexually transmitted infections including HPV infection (20, 24, 29–33). Consequently, strong associations between BV and related bacterial species with high-risk HPV infection, prevalence, persistence, and disease have been reported (17, 21, 23, 25, 27, 34–36). It is not fully clear whether changes in the CVM contribute to cervical cancer or are secondary to HPV-infection and HPV-induced neoplastic disease. However, some studies suggest that changes in CVM composition may precede HPV-induced disease development (37–40). Overall, the mechanistic and temporal interactions between the CVM and HPV pathogenesis have not been fully elucidated.

These findings highlight the need for tractable, preclinical models to study this biological interplay. Studies performed using HPV16 transgenic mice have provided some insight into this interaction (41, 42), but transgenic mice lack key features of infection-mediated neoplastic progression. A recently discovered murine papillomavirus, MmuPV1, has revolutionized the papillomavirus research field, facilitating the study of viral transmission, replication, persistence, and pathogenesis in an animal model (43–45). Several labs, including our own, have developed MmuPV1 infection-based models to study both cutaneous and mucosal papillomavirus infection and disease (46–63). In an infection-based preclinical murine model of cervicovaginal cancer, we demonstrated that MmuPV1 establishes productive viral infections and causes progressive neoplastic disease in the female reproductive tract of immunocompetent *FVB/N* mice (60). Following experimental MmuPV1 infection of the female reproductive tract, a subset of MmuPV1-infected mice spontaneously clear the infection, while others establish high viral load infections that can persist for at least 10 months (59, 64). We have also demonstrated that MmuPV1 has oncogenic potential in the skin, anus, and oral cavity of immunocompetent *FVB/N* mice (47, 61, 62, 65). Thus, MmuPV1 provides a powerful new papillomavirus infection model to investigate the role of viral as well as environmental and host factors in papillomavirus-induced cancers.

In this study, we used MmuPV1 to evaluate how papillomavirus infection, persistence, and neoplastic disease influence the host cervicovaginal microbiome in an *in vivo* preclinical murine model. Using 16S ribosomal RNA gene sequencing of DNA extracted from either cervicovaginal lavages or laser-captured regions of infected tissue, we found that key aspects of MmuPV1 infection affect the composition of both the global and local CVM, respectively, in the female reproductive tract of infected mice. We also observed significant variability in the natural host CVM in uninfected mice across experiments, and the baseline CVM present at the time of infection appeared to ultimately influence how MmuPV1 altered the overall microbial community composition with respect to specific bacterial taxa. Infection metrics identified as being important in human disease, such as viral load, viral persistence, and neoplastic disease severity, were consistently associated with changes in the overall diversity and composition of the host CVM. To our knowledge, this is the first study to define changes in the host microbiome associated with key events during infection of the reproductive tract with the recently discovered murine papillomavirus MmuPV1. Our findings represent important progress toward the long-term objective of using the MmuPV1 infection model to further explore the bidirectional interplay between the host CVM and papillomavirus infection, persistence, and malignant progression in the female reproductive tract.

## RESULTS

### The murine cervicovaginal microbiome exhibits high inter-individual variability across experiments

**Fig 1:**
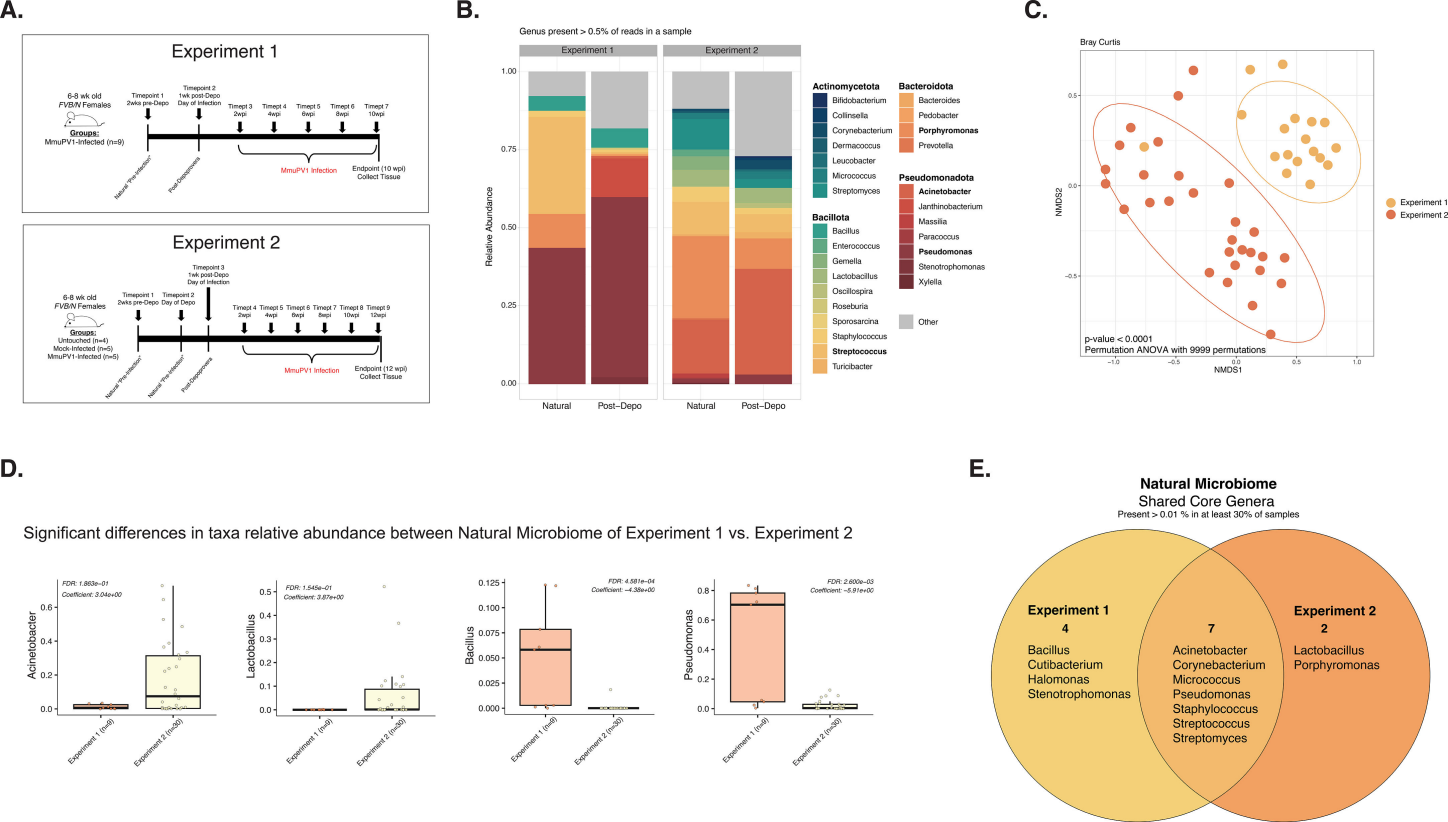
The natural murine cervicovaginal microbiome exhibits high inter-individual variability across experiments. For each experiment, DNA samples extracted from cervicovaginal lavages were sent for high-throughput sequencing of the bacterial 16S rRNA gene. (**A**) Schematics detailing the main hallmarks and timepoints sampled throughout Experiment 1 (top) and Experiment 2 (bottom). Arrows indicate times where cervicovaginal lavages were collected. Times labeled with “Timepoints” or “Timepts” indicate samples that were also screened by 16S sequencing. Numbers of mice per group are indicated. (**B**) Relative abundance of microbial genera present in at least 0.5% of the cervicovaginal microbial community for mice before any intervention (the “natural cervicovaginal microbiome”; samples from all mice in untouched, mock-infected, and MmuPV1-infected groups before infection) and 1 week after treatment with Depo-Provera (samples from all mock-infected and MmuPV1-infected mice) from both Experiments 1 and 2. (**C**) Bray-Curtis beta diversity ordination of “natural” mice from each experiment, prior to any intervention. PERMANOVA analysis was performed to determine significant differences among groups (*P*-value < 0.0001). (**D**) Microbiome Multivariable Association with Linear Models version 2 (MAASLIN2) was used to identify taxa significantly associated with each of the experiments. The *y*-axis indicates the relative abundance of the genera. For all significant comparisons, the False Discovery Rate (FDR) adjusted *P*-value with the Benjamini-Hochberg correction method along with the model coefficient value (effect size), indicating the degree of contrast between the selected category against the reference category (Experiment 1), are shown. (**E**) Venn diagram of the core-taxa analysis for the natural microbiome, representing the bacterial taxa present in >0.01% in at least 30% of the samples from mice prior to any intervention from Experiments 1 and 2.

We first sought to define the natural cervicovaginal microbiome (CVM) in uninfected female mice on the *FVB/N* genetic background, which are immunocompetent mice susceptible to MmuPV1 infection and pathogenesis (60). In two independent experiments (Experiments 1 and 2; Fig. 1A; Table 1), cervicovaginal lavages were performed on 6- to 8-week-old female *FVB/N* mice prior to experimental manipulation (natural) and the bacterial microbiome was profiled. Vaginal microbial communities from mice in Experiment 1 were dominated by taxa within the *Pseudomonas* genera*,* while communities in Experiment 2 were dominated by *Acinetobacter* and *Porphyromonas* (Fig. 1B). Bray-Curtis dissimilarity analysis further confirmed that the naturally-occurring composition of the CVMs of mice from Experiment 1 and Experiment 2 were distinct (PERMANOVA; *P*-value < 0.0001; Fig. 1C; Table 2). Multivariable association analysis using MAASLIN2 (66) revealed that these differences were, in part, driven by significantly higher proportions of *Pseudomonas* and *Bacillus* and lower *Acinetobacter* and *Lactobacillus* in mice from Experiment 1 versus Experiment 2 (FDR *q*-values with Benjamini-Hochberg correction <0.05; Fig. 1D). Despite these overall differences, there were seven core genera that were commonly represented across both experiments: *Acinetobacter*, *Corynebacterium*, *Micrococcus*, *Pseudomonas*, *Staphylococcus*, *Streptococcus*, and *Streptomyces* (Fig. 1E). These results indicate that the natural cervicovaginal microbiome of *FVB/N* mice is variable and can differ across experiments.

**TABLE 1:** Experiment overview for experiments 1–5^*a*^

Experiment	Description	Experimental groups	Full experimental time course	Microbiome assessment timepoints
Experiment 1	Natural/pre-infection and early infection	MmuPV1-infected	Natural (pre-infection), 1 wk post-Depo, 2–10 wpi	Natural (pre-infection); 1 wk post-Depo; 2, 4, 6, 8, 10, and 12 wpi
Experiment 2	Natural/pre-infection and early infection	Untouched, mock-infected, MmuPV1- infected	Natural (pre-infection), 1 wk post-Depo, 2–12 wpi	Natural (pre-infection); 1 wk post-Depo; 2, 4, 6, 8, and 10 wpi
Experiment 3	Late infection	Mock-infected, MmuPV1-infected	4.5–24 wpi	4.5, 7, 9, 14, 18, and 24 wpi
Experiment 4	Viral dose curve	Mock-infected, 10^4^ VGE, 10^6^ VGE, or 10^8^ VGE MmuPV1	2–24 wpi	8 wpi, 20 wpi
Experiment 5	Laser capture microdissection (local microbiome)	Mock-infected, mock-infected + estrogen, MmuPV1-infected, MmuPV1-infected + estrogen	24 wpi	24 wpi

^*a*^ A brief description of the experiments in this study, including experimental groups and time points where the microbiome was assessed. (wpi, weeks post-infection; wk, week).

**TABLE 2:** Permanova analyses^*a*^^*,^b^*^

Experiment	Groups included	Variable	Variable levels	R2	P-value	P-value significance	q-value	q-value significance	Notes
Experiment 1	All (infected mice)	Pre- v. Post-Infection	Pre-Infection v. Post-Infection	0.04829	1.30E-02	*	0.0303	*	
Experiment 1	All (infected mice)	Infection Stage (pre-infect v. post-depo v. infection establishment v. early infection v. mid infection)	Pre-Infection, Post-Depoprovera, Infection Establishment, Early Infection, Mid Infection	0.3	1.00E-04	***	0.0004	***	
Experiment 1	All (infected mice)	Viral copy number	Low v. High	0.0573	0.0590	°	0.1033	ns	
Experiment 1	All (infected mice)	Viral Persistence	Persistence	--	--	--	--	--	All infected mice had viral persistence
Experiment 1	All (infected mice)	Cervicovaginal Dysplasia Stage	CIN 2, CIN 3, SCC	0.0777	0.2330	ns	0.3262	ns	
Experiment 1	All mice (post infection timepoints only)	Infection Stage (infection establishment v. early infection v. mid infection)	Pre-Infection, Post-Depoprovera, Infection Establishment, Early Infection, Mid Infection	0.3169	1.00E-04	***	0.0004	***	
Experiment 1	All mice (post infection timepoints only)	Viral copy number (H vs. L)	Low v. High	0.0151	0.6657	ns	0.7767	ns	
Experiment 1	All mice (post infection timepoints only)	Viral Persistence	Persistence	--	--	--	--	--	All infected mice had viral persistence
Experiment 1	All mice (post infection timepoints only)	Cervicovaginal Dysplasia Stage	CIN 2, CIN 3, SCC	0.0493	0.7972	ns	0.7972	ns	
Experiment 2	All mice	Intervention Group	Untreated, Mock Infected, mMUPV1 Infected.	0.0316	0.0293	*	0.0553	°	
Experiment 2	All mice	Pre- vs. Post-Infection	Pre-Infection v. Post-Infection	0.04178	1.00E-04	***	0.0006	***	
Experiment 2	All mice	Pre-Infection vs. Post-infection Untouched, Mock, and Infected	Pre-Infection, Post-Depoprovera, Untouched, Mock, Infected (at post-infection timepoints)	0.10989	1.00E-04	***	0.0006	***	
Experiment 2	All mice	Infection Stage	Pre-Infection, Post-Depoprovera, Infection Establishment, Early Infection, Mid Infection	0.04178	1.00E-04	***	0.0006	***	
Experiment 2	All mice	Viral copy number	Low v. High	0.01861	0.1981	ns	0.2806	ns	
Experiment 2	All mice	Viral Persistence	Persistence	--	--	--	--	--	All infected mice had viral persistence
Experiment 2	All mice	Cervicovaginal Dysplasia Stage	CIN 2, CIN 3	--	--	--	--	--	Only histology from Infected mice was scored. No histology from Untouched or Mock infected mice.
Experiment 2	All mice (post infection timepoints only)	Intervention Group	Untreated, Mock Infected, mMUPV1 Infected.	0.0599	0.0018	**	0.0061	**	
Experiment 2	Untreated Mice	Pre- vs. Post-Infection	Pre-Infection v. Post-Infection	0.0253	0.5674	ns	0.6029	ns	
Experiment 2	Untreated Mice	Infection Stage	Pre-Infection, Post-Depoprovera, Infection Establishment, Early Infection, Mid Infection	0.1288	0.5421	ns	0.6029	ns	
Experiment 2	Mock infection mice	Pre- vs. Post-Infection	Pre-Infection v. Post-Infection	0.0887	0.0013	**	0.0055	**	
Experiment 2	Mock infection mice	Infection Stage	Pre-Infection, Post-Depoprovera, Infection Establishment, Early Infection, Mid Infection	0.1751	0.0054	**	0.0131	*	
Experiment 2	Infected Mice	Pre- vs. Post-Infection	Pre-Infection v. Post-Infection	0.0606	0.0031	**	0.0089	**	
Experiment 2	Infected Mice	Infection Stage	Pre-Infection, Post-Depoprovera, Infection Establishment, Early Infection, Mid Infection	0.1483	0.0099	**	0.021	*	
Experiment 2	Infected Mice	Viral copy number	Low v. High	0.0609	0.1925	ns	0.2806	ns	
Experiment 2	Infected Mice	Viral Persistence	Persistence	--	--	--	--	--	All infected mice had viral persistence
Experiment 2	Infected Mice	Cervicovaginal Dysplasia Stage	CIN 2, CIN 3	0.0945	0.683	ns	0.683	ns	
Experiment 2	Infected Mice (post infection Only)	Infection Stage	Infection Establishment, Early Infection, Mid Infection	0.09341	0.3099	ns	0.3763	ns	
Experiment 2	Infected Mice (post infection Only)	Viral copy number	Low v. High	0.0726	0.3099	ns	0.3763	ns	
Experiment 2	Infected Mice (post infection Only)	Viral Persistence	Persistence	--	--	--	--	--	All infected mice had viral persistence
Experiment 2	Infected Mice (post infection Only)	Cervicovaginal Dysplasia Stage	CIN 2, CIN 3	0.1718	0.133	ns	0.226	ns	
Experiment 3 (late Infection)	All	Mock v. Infection	Mock v. MmuPV1 infection	0.0155	0.3943	ns	0.4929	ns	
Experiment 3 (late Infection)	All	Infection Stage	Early Infection, Mid infection, Late Infection	0.058	0.0054	**	0.043	*	
Experiment 3 (late Infection)	All	Viral copy number	Low v. High	0.0494	0.3046	ns	0.4929	ns	
Experiment 3 (late Infection)	All	Viral Persistence	Persistence v. Clearance	0.0441	0.567	ns	0.63	ns	
Experiment 3 (late Infection)	All	Cervicovaginal Dysplasia Stage	Normal to hyperplasia, CIN 3, CIN 3+	0.0382	0.141	ns	0.3525	ns	
Experiment 3 (late Infection)	Infected Mice	Infection Stage	Early Infection, Mid infection, Late Infection	0.0651	0.0086	**	0.043	*	
Experiment 3 (late Infection)	Infected Mice	Viral copy number	Low v. High	0.0398	0.317	ns	0.4929	ns	
Experiment 3 (late Infection)	Infected Mice	Viral Persistence	Persistence v. Clearance	0.0346	0.6952	ns	0.6952	ns	
Experiment 3 (late Infection)	Infected Mice	Cervicovaginal Dysplasia Stage	CIN 3, CIN 3+	0.0385	0.3735	ns	0.4929	ns	
Experiment 3 (late Infection)	Mock infection mice	Infection Stage	Early Infection, Mid infection, Late Infection	0.0651	0.0666	°	0.222	ns	
Experiment 4 (Viral Dose)	All	Viral Inoculation Dose	Mock, 10^4, 10^6, 10^8	0.165	0.0034	**	0.0059	**	
Experiment 4 (Viral Dose)	All	Viral copy number	Low v. High	0.1148	0.0044	**	0.0059	**	
Experiment 4 (Viral Dose)	All	Viral Persistence	Persistence v. Clearance	0.1432	9.00E-04	***	0.0024	**	
Experiment 4 (Viral Dose)	All	Cervicovaginal Dysplasia Stage	Normal, CIN1, CIN2, CIN 3, SCC	0.13389	0.37	ns	0.37	ns	
Experiment 4 (Viral Dose)	Infected Mice	Viral Inoculation Dose	Mock, 10^4, 10^6, 10^8	0.1253	0.0041	**	0.0059	**	
Experiment 4 (Viral Dose)	Infected Mice	Viral copy number	Low v. High	0.11326	1.00E-04	***	0.0008	***	
Experiment 4 (Viral Dose)	Infected Mice	Viral Persistence	Persistence v. Clearance	0.1014	4.00E-04	***	0.0016	**	
Experiment 4 (Viral Dose)	Infected Mice	Cervicovaginal Dysplasia Stage	Normal, CIN1, CIN2, CIN 3, SCC	0.1854	0.0276	*	0.0315	*	
Experiment 5 (LCM)	All	Group	Mock, Mock + estrogen, Infected, Infected + estrogen	0.901	0.0624	°	0.3266	ns	
Experiment 5 (LCM)	All	Mock v. Infection	Mock v. MmuPV1 infection	0.0315	0.0994	°	0.3266	ns	
Experiment 5 (LCM)	All	Estrogen Supplementation	Estrogen v. No Estrogen	0.0339	0.0657	°	0.3266	ns	
Experiment 5 (LCM)	All	Estrus stage	proestrus, estrus, metestrus, diestrus	0.1189	0.9889	ns	0.9911	ns	
Experiment 5 (LCM)	All	Sampling Location	Ectocervix, Cervicovaginal wall, Vaginal wall	0.0721	0.9844	ns	0.9911	ns	
Experiment 5 (LCM)	All	Cervicovaginal Dysplasia Stage	Normal to hyperplasia, CIN 2, CIN 3, SCC	0.1054	0.2318	ns	0.6544	ns	
Experiment 5 (LCM)	All Mock infected mice	Estrogen Supplementation	Estrogen v. No Estrogen	0.0473	0.6681	ns	0.9207	ns	
Experiment 5 (LCM)	All Mock infected mice	Estrus stage	proestrus, estrus, metestrus, diestrus	0.2175	0.3416	ns	0.7163	ns	
Experiment 5 (LCM)	All Mock infected mice	Sampling Location	Ectocervix, Cervicovaginal wall, Vaginal wall	0.0553	0.3426	ns	0.7163	ns	
Experiment 5 (LCM)	All Infected Mice	Estrogen	Estrogen v. No Estrogen	0.0702	0.0527	°	0.3266	ns	
Experiment 5 (LCM)	All Infected Mice	Estrus stage	proestrus, estrus, metestrus, diestrus	0.1743	0.897	ns	0.9911	ns	
Experiment 5 (LCM)	All Infected Mice	Sampling Location	Ectocervix, Cervicovaginal wall, Vaginal wall	0.1449	0.4191	ns	0.7415	ns	
Experiment 5 (LCM)	All Infected Mice	Viral copy number	Low v. High	0.067	0.0788	°	0.3266	ns	
Experiment 5 (LCM)	All Infected Mice	Viral Persistence	Persistence	--	--	--	--	--	All infected mice had viral persistence
Experiment 5 (LCM)	All Infected Mice	Cervicovaginal Dysplasia Stage	Normal to hyperplasia, CIN 2, CIN 3, SCC	0.097	0.4064	ns	0.7415	ns	
Experiment 5 (LCM)	No Estrogen Mice	Mock v. Infection	Mock v. MmuPV1 infection	0.07	0.5519	ns	0.8772	ns	
Experiment 5 (LCM)	No Estrogen Mice	Estrus stage	proestrus, estrus, metestrus, diestrus	0.2264	0.9911	ns	0.9911	ns	
Experiment 5 (LCM)	No Estrogen Mice	Sampling Location	Ectocervix, Cervicovaginal wall, Vaginal wall	0.103	0.6805	ns	0.9207	ns	
Experiment 5 (LCM)	No Estrogen + Infected	Viral copy number	Low v. High	0.1198	0.8571	ns	0.9911	ns	note: two had low viral load
Experiment 5 (LCM)	No Estrogen + Infected	Cervicovaginal Dysplasia Stage	CIN 3, CIN 3	0.2305	0.0952	°	0.3266	ns	
Experiment 5 (LCM)	Estrogen Mice	Mock v. Infection	Mock v. MmuPV1 infection	0.0534	0.0444	*	0.3266	ns	
Experiment 5 (LCM)	Estrogen Mice	Estrus stage	proestrus, estrus, metestrus, diestrus	0.1214	0.9623	ns	0.9911	ns	
Experiment 5 (LCM)	Estrogen Mice	Sampling Location	Ectocervix, Cervicovaginal wall, Vaginal wall	0.2215	0.5721	ns	0.8772	ns	
Experiment 5 (LCM)	Estrogen + Infection	Viral copy number	Low v. High	--	--	--	--	--	Only one had Low viral load so unable to assess
Experiment 5 (LCM)	Estrogen + Infection	Cervicovaginal Dysplasia Stage	CIN 2, CIN 3, SCC	0.1594	0.2561	ns	0.6545	ns	

^*a*^ The Bray-Curtis metric was utilized to assess beta-diversity between cervicovaginal microbiome samples for each of the experiments included in this study. Univariate permutation ANOVAS with 9999 permutations were then used to assess whether sample microbial community beta diversity significantly clustered by intervention group (i.e., mock-infected vs MmuPV1-infected) or infection outcomes (i.e., high vs low viral load, cervical dysplasia severity, etc.). The table summarizes the permutation ANOVA results for each analysis within each experiment.

^*b*^ To account for multiple comparisons, significance values were corrected with the Benjamini-Hochberg (B-H) Procedure. LCM: Laser capture microdissection.ns: not significant.-- indicates unable to assess. See “Notes” column for details.° indicates the p-value or the B-H adjusted q-value < 0.1, * indicates the p-value or the B-H adjusted q-value < 0.05, ** indicates the p-value or the B-H adjusted q-value < 0.01, *** indicates the p-value or the B-H adjusted q-value < 0.001.

All mock-infected and MmuPV1-infected mice in these studies were pre-treated with medroxyprogesterone acetate (otherwise known by the brand name Depo-Provera or “Depo”), which is used to synchronize mice in the diestrus phase of the estrus cycle when the cervicovaginal epithelia is thinnest (67) and thus more susceptible to papillomavirus infection. The changes and extent to which Depo-Provera treatment influenced CVM composition were specific to each experiment (Fig. S1A and B) with a group of eight core taxa changed across both experiments (Fig. S1C). Together, these results indicate that the host murine cervicovaginal microbiome in female *FVB/N* mice is naturally variable in its composition and is likewise altered in a host- and experiment-specific manner after administration of Depo-Provera in this MmuPV1 infection model. Because of these findings, we elected to analyze subsequent experiments individually.

### MmuPV1 infection shapes murine cervicovaginal microbial community composition

**Fig 2:**
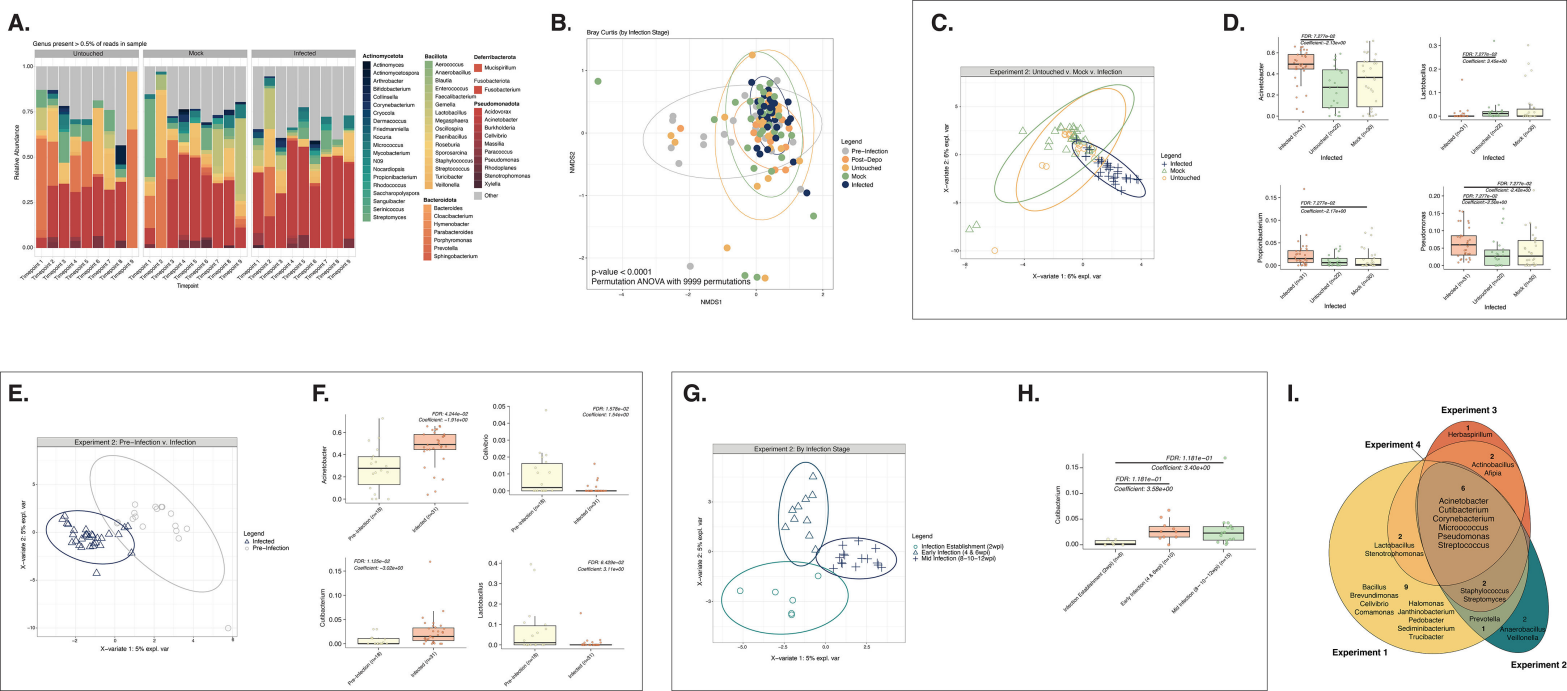
MmuPV1 infection shapes host cervicovaginal microbial community composition. All plots in this figure except part 2I represent mice from Experiment 2. (**A**) Relative abundance of microbial genera present in at least 0.5% of the cervicovaginal microbial community for untreated, mock-infected, and MmuPV1-infected mice. Each bar represents the average relative abundance for mice within each group at each timepoint. Timepoints range from pre-intervention (Timepoints 1 and 2; the “natural community”), 1 week post-Depo-Provera (Timepoint 3), and 2–12 weeks post-infection (wpi) (Timepoints 4–9). (**B**) Bray-Curtis beta diversity ordination of cervicovaginal microbial community composition grouped by pre-infection, post-depo, and the untouched, mock infected, and infected groups at post-infection timepoints. PERMANOVA analysis was performed to determine significant differences among groups (*P*-value < 0.0001). (**C**) Supervised PLS-DA ordination distinguishing cervicovaginal microbial communities from untouched, mock-infected, and MmuPV1-infected mice. A corresponding vector plot indicating key microbial genera that help distinguish each of the groups in the supervised ordination is shown in Fig. S2A. (**D**) MAASLIN2 plots indicating genera significantly more or less abundant in MmuPV1-infected mice compared to mock-infected and/or untouched mice from Experiment 2 (Fig. 2). Note, no significant differences were observed between the mock-infected and untouched mice via MAASLIN2. (**E**) Supervised PLS-DA ordination distinguishing samples from pre-infection or post-infection cervicovaginal microbiomes. A corresponding vector plot indicating key microbial genera that help distinguish each of the groups in the supervised ordination is shown in Fig. S2B. (**F**) Genera significantly more or less abundant before (pre-infection; Timepoints 1–3) or after MmuPV1 infection (Timepoints 4–9) via MAASLIN2. (**G**) Supervised PLS-DA ordination distinguishing cervicovaginal microbial communities during infection establishment (2 wpi), early infection (4–6 wpi), and mid infection (8–10–12 wpi). The corresponding vector plot, indicating key microbial genera that help distinguish each of the groups in the supervised ordination, is shown in Fig. S2C. Similar analyses to these for the mice in Experiment 1, all of which were infected with MmuPV1, and Experiment 3, mock-infected and MmuPV1-infected mice 5–25 wpi, are displayed in Fig. S2D through H ,I–L, respectively. (**H**) Genera significantly more or less abundant in mouse cervicovaginal communities during infection establishment, early infection, or mid-infection via MAASLIN2. (**I**) Venn diagram of shared core taxa in mouse cervicovaginal microbial communities following MmuPV1 infection across Experiments 1–4 in this study. Lavages were used to sample cervicovaginal microbial communities for all experiments shown. Diagram indicates taxa present in >0.01% in at least 30% of the samples. Due to distinct differences in background cervicovaginal microbial communities, mice from each experiment were assessed separately. Additional analyses on mice from Experiment 1 and Experiment 3 (mid-late infection) are represented in Plots D–H and I–L, respectively, in Fig. S2.

To determine the overall effects of papillomavirus infection on the host CVM over time, we collected cervicovaginal lavages from untouched, mock-infected, and/or infected mice over the course of 12 weeks following infection with 10^8^ viral genome equivalents (VGE) of MmuPV1 (Experiment 2; Fig. 1A bottom; Table 1) and performed 16S sequencing. Figure 2A illustrates the relative abundance of bacterial genera within the CVM of mice in each group over the course of Experiment 2. Timepoints 1–2 represent the natural/pre-infection microbiome, Timepoint 3 represents the microbiome 1 week post-Depo (for the mock- and MmuPV1-infected mice), and Timepoints 4–9 represent 2–12 weeks after MmuPV1 infection, mock infection, or no intervention.

We evaluated the CVM across all groups in Experiment 2 using unsupervised Bray-Curtis analysis (Fig. 2B). We found that microbial community composition differed between samples collected from all mice at pre-infection timepoints, samples collected from mock- and MmuPV1-infected mice 1 week post-Depo, and samples collected longitudinally from untouched, mock-infected, and MmuPV1-infected groups at post-infection timepoints (PERMANOVA; *P*-value < 0.0001; Table 2). When we limited our analysis to samples taken from all mice at Timepoints 2–12 weeks post-infection, microbial community composition of the untouched, mock-infected, and MmuPV1-infected groups of mice was also distinct (PERMANOVA *P*-value < 0.01; Table 2). To predict key microbial genera that help distinguish cervicovaginal samples from different conditions, microbial data sets were incorporated into supervised Partial Least Squares-Discriminant Analyses (PLS-DA). The PLS-DA ordination plot illustrates the relationship and similarities of each sample based on the results of the PLS-DA model and accompanying vector plots display the key microbial taxa driving differences between each group. The longer the vector, the greater the influence the taxa have in driving the separation. Both PLS-DA analysis (ordination plot in Fig. 2C; companion vector plot in Supplemental 2A) and MAASLIN2 (Fig. 2D) found that MmuPV1 infection was associated with increased *Acinetobacter* and *Pseudomonas* as well as reduced *Lactobacillus* (MAASLIN2; FDR *q*-values < 0.1). When we compared samples taken from the same mice, either before or after MmuPV1 infection, there were differences in pre- and post-infection microbial community structure via Bray-Curtis dissimilarity (PERMANOVA analysis; *P*-value < 0.01, Table 2). Corresponding PLS-DA analysis revealed *Cellvibrio* and *Lactobacillus* to be among the taxa driving pre-infection sample clustering, whereas *Acinetobacter* and *Cutibacterium* were among the main taxa driving MmuPV1-infected sample clustering (Fig. 2E; Fig. S2B). These taxa were also identified via MAASLIN2 analysis (Fig. 2F). Likewise, there were significant differences in the CVM between pre-infection and MmuPV1-infected groups in Experiment 1 (PERMANOVA analysis; *P*-value < 0.05; Table 2). Differences between mock-infected and MmuPV1-infected groups in Experiment 3 did not reach statistical significance. Collectively, these results provide evidence that MmuPV1 infection can influence the host cervicovaginal microbiome although the specific top driving taxa differ across experiments (Fig. 2C through D [Experiment 2], Fig. S2D through F [Experiment 1] and Fig. S2I through J [Experiment 3].

Our longitudinal analyses also allowed us to evaluate whether temporal stages of MmuPV1 infection affect the host CVM. We compared samples collected from Experiment 2 during MmuPV1 infection establishment (2 wpi), early infection (4–6 wpi) and mid-infection (8–12 wpi). Overall, CVM composition was significantly different across these stages (PERMANOVA analysis; *P*-value < 0.0001; Table 2). While several driving taxa were identified using PLS-DA analysis that distinguish infection stages (Fig. 2G; Fig. S2C), MAASLIN2 analysis only identified the genus *Cutibacterium* as being in higher relative abundance at later stages of MmuPV1 infection (Fig. 2H). We also saw differences in the microbiome across infection stages in Experiment 1 (Fig. S2G through H; PERMANOVA; *P*-value < 0.0001, Table 2) and Experiment 3 (Fig. S2K through L; PERMANOVA; *P*-value < 0.01; Table 2). Considering these data across multiple experiments, we conclude that MmuPV1 infection influences the host CVM in a manner that changes over time as the infection proceeds. However, the exact manner and extent to which MmuPV1 infection influences the host microbiome differed by experiment.

Across four independent experiments, six core microbial taxa were shared in mice infected with MmuPV1: *Acinetobacter, Corynebacterium, Cutibacterium, Micrococcus, Pseudomonas,* and *Streptococcus* (Fig. 2I). However, 12 taxa were unique to only infected mice from one experiment. Collectively, these observations provide additional lines of evidence that the predominant natural CVM is highly variable across experiments and suggest that the baseline host CVM community composition likely sets the trajectory of microbiome changes induced by MmuPV1 infection.

### MmuPV1 inoculation dose and viral persistence influences infection outcomes and cervicovaginal microbial communities

**Fig 3:**
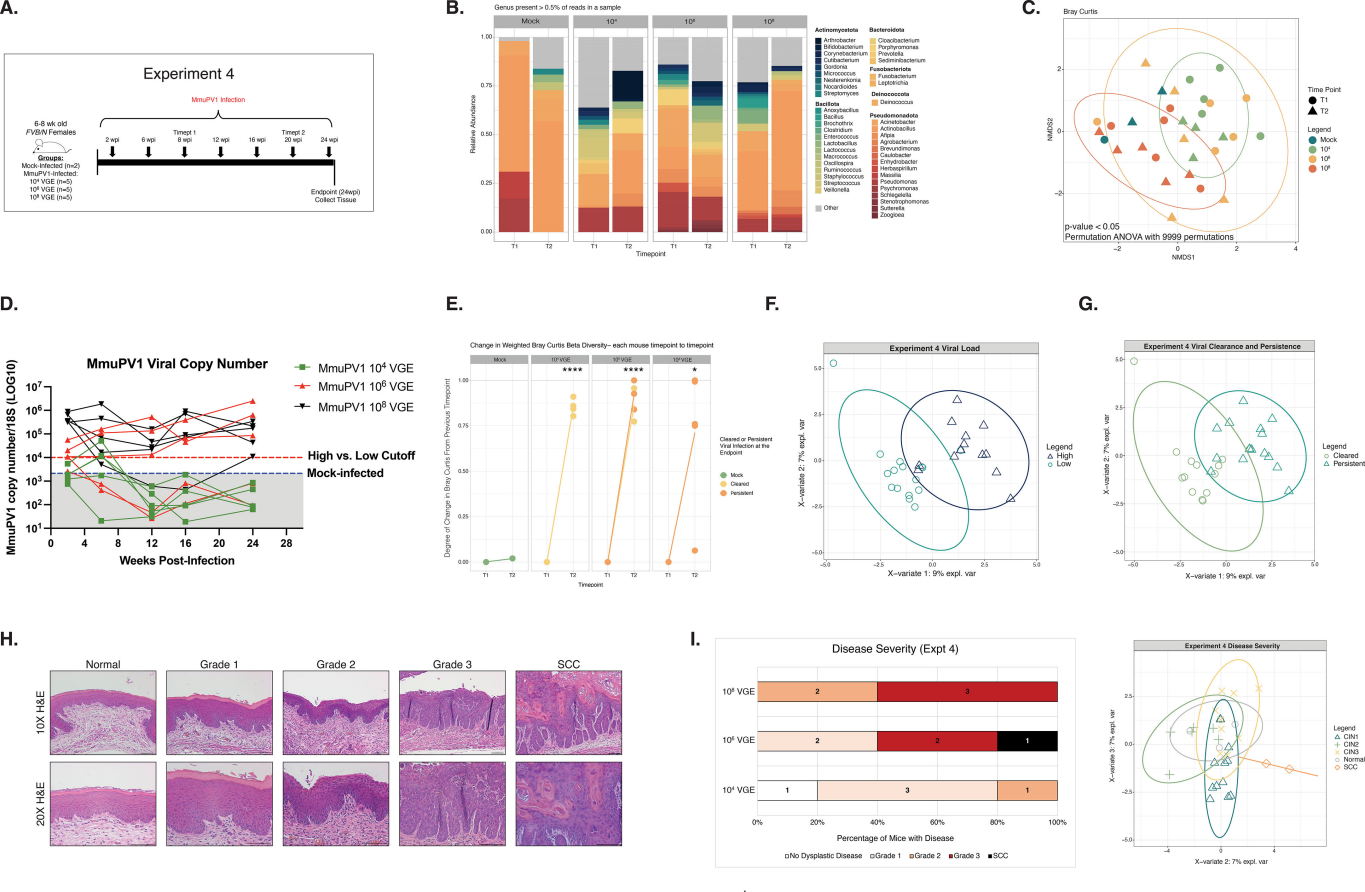
MmuPV1 inoculation dose and viral persistence influences infection outcomes and cervicovaginal microbial communities. (**A**) A schematic detailing the main hallmarks and timepoints sampled throughout Experiment 4 in which mice were initially either mock-infected or inoculated with 10^4^, 10^6^, or 10^8^ viral genome equivalents (VGE). Arrows indicate times where cervicovaginal lavages were collected for measuring MmuPV1 copy number and/or assessing cervicovaginal microbial community composition. Times labeled with “Timepoints” or “Timepts” indicate samples that were also screened by 16S sequencing. Numbers of mice per group are indicated. (**B**) Relative abundance of microbial genera present in at least 0.5% of mouse cervicovaginal microbial communities assessed at 8 and 20 weeks post-infection (wpi). Each bar represents the average relative abundance for mice within that group at each timepoint. (**C**) Bray-Curtis beta diversity ordination. Colors indicate viral inoculation group and shape indicates the timepoint of cervicovaginal microbiome samples. PERMANOVA analysis was performed to determine significant differences among groups (*P*-value < 0.05). (**D**) At multiple timepoints indicated in 3A, cervicovaginal lavages were collected, DNA extracted, and qPCR performed for the MmuPV1 E2 gene to quantify viral copy numbers. Infected mice with viral copy numbers greater than 1 × 10^4^ copies were considered to have high viral load (red line). Values detected in mock-infected mice represent background levels (2.13 × 10^3^; blue line). (**E**) Change in weighted Bray-Curtis Beta Diversity between 8 wpi (Timepoint 1; **T1**) and 20 wpi (Timepoint 2; **T2**) in mock-infected mice and mice infected with doses of 10^4^, 10^6^, or 10^8^ VGE MmuPV1. Lines associated with those mice that cleared MmuPV1 infections as determined by viral load qPCR measurements (Fig. 3D) are shaded in yellow, and mice that developed persistent MmuPV1 infections are shaded in orange. (**F**) Supervised PLS-DA ordination plot distinguishing cervicovaginal microbial communities from mice initially inoculated with either 10^4^ or 10^8^ VGE that achieved either high or low viral load. A companion vector plot is shown in Fig. S3C. (**G**) Supervised PLS-DA distinguishing cervicovaginal microbial communities from mice who had viral persistence or clearance at the end point. A companion vector plot is shown in Fig. S3E. No significant differences were observed between mice with viral persistence or viral clearance via MAASLIN2 analysis. (**H**) At the conclusion of each experiment, female reproductive tract tissues were collected and H&E-stained sections used to perform histopathological analysis. Overall disease severity was scored by a trained pathologist (SMM) and worst disease reported for each tissue sample. Representative images (10× magnification, bottom; 20× magnification, top) of H&E-stained sections are shown for normal pathology, stages of progressive neoplastic disease ranging from Grade 1 to Grade 3 dysplasia, and squamous cell carcinoma (SCC). All scale bars = 100 µm. (**I**) Bar graph showing overall disease severity measured by histopathological analysis at the 24 wpi study endpoint of Experiment 4 for groups of mice inoculated with different doses of MmuPV1 (10^4^, 10^6^, or 10^8^ VGE; left). The plot on the right shows the supervised PLS-DA ordination distinguishing cervicovaginal microbial communities from mice with no disease or hyperplasia (normal) or with various stages of cervical neoplastic disease: Grade 1/CIN1, Grade 2/CIN2, Grade 3/CIN3, or squamous cell carcinoma (SCC). For each mouse, both post-infection timepoints were included in the PLS-DA and points are colored by the cervical dysplasia score at the endpoint. Figure S3G contains the companion vector plot. No significant differences were observed between disease severity groups via MAASLIN2.

In humans, high viral loads are highly predictive of viral persistence and disease development (10–12, 68–71). We have observed similar associations between viral load and disease severity in the MmuPV1 cervicovaginal infection model (60, 64). Therefore, we next evaluated whether MmuPV1 viral load and neoplastic disease severity are associated with changes in the host CVM. To do so, we performed a MmuPV1 dose curve study to experimentally regulate MmuPV1 viral load, viral clearance/persistence, and neoplastic disease development. Groups of mice were inoculated with either 10^4^, 10^6^, or our standard dose of 10^8^ VGE per mouse and cervicovaginal lavages were collected at times post-MmuPV1 infection between 2 and 24 wpi (Fig. 3A; Table 1). We profiled the microbiome of mock-infected and MmuPV1-infected samples collected at 8 wpi (Timepoint 1) and 20 wpi (Timepoint 2) (Fig. 3B). The Bray-Curtis dissimilarity of microbial communities present across these groups was statistically distinct (PERMANOVA analysis *P*-value < 0.01; Fig. 3C; Table 2), with the 10^8^ VGE dose showing the greatest separation. PLS-DA analysis identified key taxa associated with each infection condition, including *Actinobacillus* (mock-infected), *Odoribacter* (10^4^ VGE), *Lactobacillus* (10^6^ VGE), and *Stenotrophomonas* (10^8^ VGE) (Fig. S3A). MAASLIN2 analysis was consistent with a greater proportion of *Actinobacillus* in the mock-infected group (FDR *q*-value = 0.2; Mock vs 10^6^ VGE MmuPV1-infected group; Fig. S3B).

Longitudinal tracking of MmuPV1 viral copy number by qPCR revealed an inoculation dose-dependent effect on subsequent viral load and viral persistence. By the 24 week endpoint, none of the mice originally inoculated with 10^4^ VGE MmuPV1 (*n* = 0/5) developed high viral load or persistent viral infections (defined by viral load measurement greater than the average value measured in mock-infected mice), while 60% of mice inoculated with 10^6^ VGE (*n* = 3/5) and 100% of mice infected with 10^8^ VGE (*n* = 5/5) established high viral-load persistent infections (Fig. 3D). Regardless of inoculation dose, all MmuPV1-infected groups displayed much larger shifts in microbial community composition between Timepoint 1 (8 wpi) and Timepoint 2 (20 wpi) compared to mock-infected mice (Fig. 3E; all *P*-values < 0.05, MmuPV1-infected group vs mock, unpaired *t*-test with welches correction). We compared mice initially inoculated with either 10^4^ or 10^8^ VGE using supervised PLS-DA ordination, which showed a divergence between CVM communities that achieved either high or low viral loads (Fig. 3F; Fig. S3C). Using MAASLIN2 analysis, higher levels of *Actinobacillus* and *Enhydrobacter* and lower levels of *Pseudomonas* and *Streptomyces* were measured in the CVMs of mice with high viral load compared to those with low viral load (Fig. S3D). PLS-DA ordination also identified changes to the host CVM based on high vs low viral loads achieved naturally during MmuPV1 infection (i.e., not by experimental dose) in additional experiments (Experiment 1: Fig. S4A through B; Experiment 3: Fig. S4F through G) although the specific taxa altered by viral load largely differed across experiments.

There were also differences detected between microbial communities in mice that cleared MmuPV1 infection compared to those that established persistent viral infections in Experiment 4 (PERMANOVA *P*-value < 0.001; Table 2) despite both groups of mice having similar changes in microbial community diversity between early and late times post-infection (Fig. 3E). In both Experiment 4 (Fig. 3G) and Experiment 3 (Fig. S3F), supervised PLS-DA analysis identified *Cutibacterium* as a taxa associated with viral clearance. Interestingly, in Experiment 4, greater abundance of *Lactobacillus* was also identified as being associated with viral clearance (Fig. S3E). Taken together, these findings indicate that MmuPV1 viral load and persistence may influence the composition of the host cervicovaginal microbiome. It is also possible that the composition of the host CVM contributes to these viral readouts. For example, it is conceivable that host inflammatory responses to MmuPV1 infection could shape the CVM in a manner that influences viral load, persistence, and other aspects of papillomavirus infection natural history in the female reproductive tract.

The Experiment 4 dose curve study resulted in a broad range of viral loads and persistence. Because MmuPV1 viral load and persistence often correlate with disease outcomes (60, 64), we performed histopathological analysis on tissues collected at the study endpoint to diagnose disease severity in MmuPV1-infected mice, which can range from Normal to Grades 1–3 of cervical dysplasia (also known as cervical intraepithelial neoplasia or CIN) to squamous cell carcinoma (SCC) (Fig. 3H), to determine the global composition of cervicovaginal microbial communities with respect to disease state. All mice inoculated with 10^8^ VGE developed moderate to severe dysplasia (Grade 2 and 3), and mice inoculated with 10^6^ VGE developed low grade (Grade 1), severe dysplasia (Grade 3) and, unexpectedly, one mouse developed cancer (SCC). However, mice infected with 10^4^ VGE either did not develop disease or exhibited low to moderate dysplasia (Grade 1 and Grade 2) (Fig. 3I, left). When we compared the microbial communities in these mice across both timepoints, we found that there was a significant difference among disease states (PERMANOVA analysis; *P*-value < 0.05; Table 2). Although PLS-DA analysis identified certain taxa driving clustering by disease state (Fig. 3I right; Fig. S3G), MAASLIN2 analysis did not identify any microbial genera that significantly differed. We also evaluated the effect of viral load and disease severity in two additional experiments (Experiments 1 and 3). Most MmuPV1-infected mice in these experiments achieved high viral load and similar disease severity scores (Fig. S4A, C, F and H). Due to the insufficient range of viral load and disease severity values, we did not detect any significant changes in microbial community structure by Bray-Curtis dissimilarity assessment in Experiments 1 and 3 (Table 2). However, supervised PLS-DA analyses detected several driving taxa across groups based on viral load and disease severity (Experiment 1: Fig. S4B and D; Experiment 3: Fig. S4G and I). Taken together, we conclude that MmuPV1 viral load, persistence, and subsequent neoplastic disease state influence the global host cervicovaginal microbiome. However, based on our findings that changes induced by MmuPV1 infection are likely dependent on the baseline community composition (Fig. 2), we hypothesize that their specific effects will also be variable across experiments.

### Influence of MmuPV1 infection and neoplastic disease states on the local cervicovaginal microbiome

**Fig 4:**
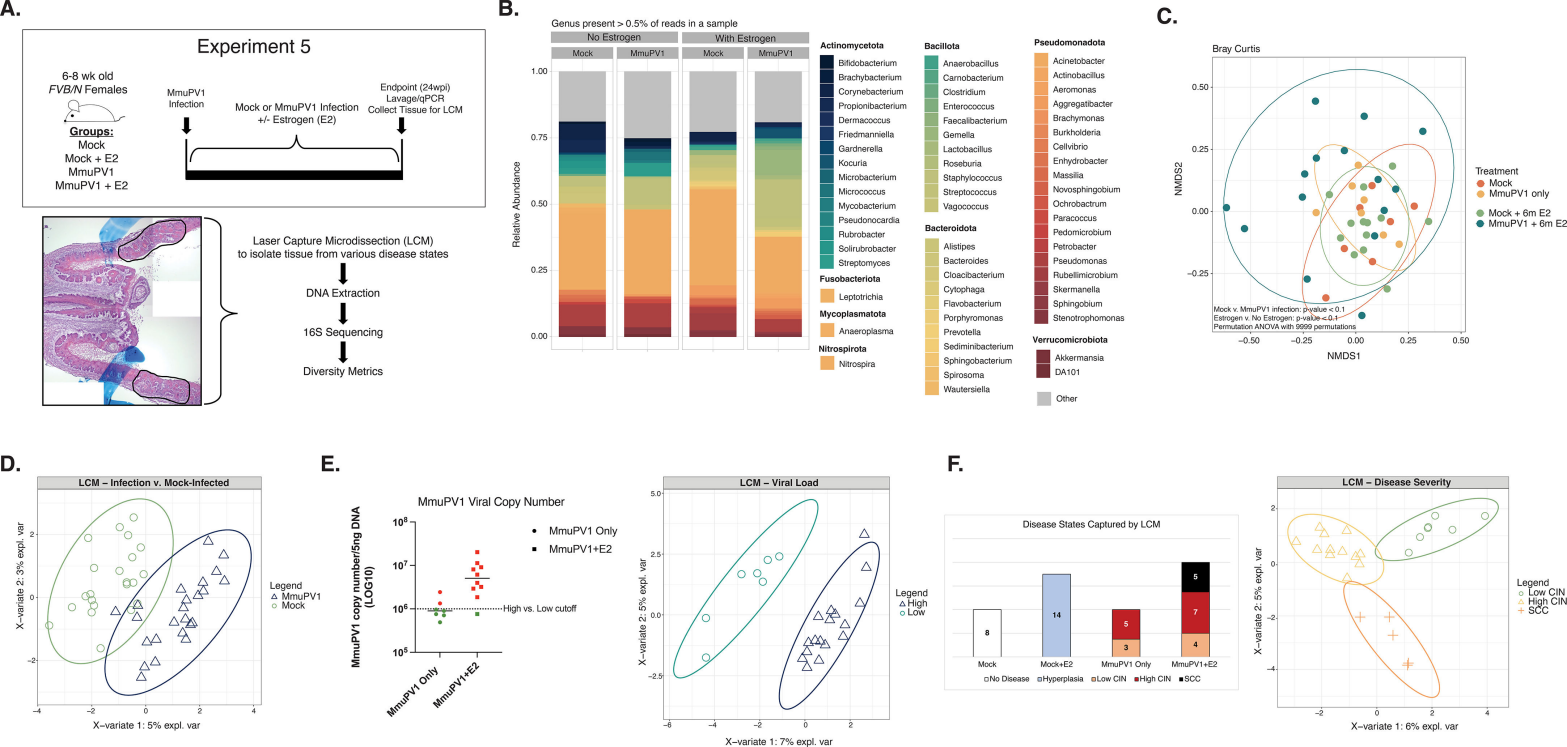
Influence of MmuPV1 infection and neoplastic disease severity on the local cervicovaginal microbiome. (**A**) Schematic of the experimental approach to studying the local cervicovaginal microbiome in MmuPV1-infected mice across different disease states (top). At the 24 wpi endpoint, lavages were performed to measure viral load by qPCR, tissues were collected, frozen in OCT, and cryosectioned. Histopathological analysis was performed to score for neoplastic disease and to identify discrete regions of each disease state, which were marked for subsequent laser capture microdissection (LCM). A representative H&E-stained tissue section with an area of disease marked for LCM is shown (blue markings: pathology markings identifying disease), along with an overview of the LCM experiment workflow. The H&E image was generated from multiple low-magnification images that were stitched, resulting in multiple background shades present in the final image. (**B**) Relative abundance of microbial genera present in at least 0.5% of mouse local cervicovaginal microbial communities. Each bar represents the average relative abundance for mice within that group. (**C**) Bray-Curtis beta diversity ordination. Samples are colored by treatment group. (**D**) Supervised PLS-DA ordination separating cervicovaginal microbial communities from laser capture-microdissected regions from mock-infected versus MmuPV1-infected mice. All mice are included in this plot regardless of estrogen treatment status. The companion vector plot is displayed in Fig. S5A. (**E**) MmuPV1 viral copy numbers measured by qPCR from cervicovaginal lavage DNA collected at the study endpoint from mice included in the LCM experiment (left). Infected mice with viral copy numbers greater than 1 × 10^6^ copies (dotted line) were considered to have high viral load (red) compared to low viral load (green). Supervised PLS-DA ordination distinguishing local microbiomes from MmuPV1-infected mice with low versus high viral load (right). The corresponding vector plot is in Fig. S5D. (**F**) Overview of disease states captured by LCM by treatment group (left). Numbers represent the number of individual LCM samples per condition. Supervised PLS-DA ordination distinguishing local cervicovaginal microbiomes from MmuPV1-infected mice based on the degree of endpoint disease severity of the laser-captured tissue (low grade dysplasia/CIN, high grade dysplasia/CIN, or SCC; left). Figure S5E contains the corresponding vector plot.

Our studies thus far have compared the microbial communities present across different neoplastic disease states by collecting cellular material sloughed from the entire cervicovaginal canal during lavages of tissues that harbor a given disease state. To determine whether the local microbiome differs within discrete regions of disease, we performed laser capture microdissection (LCM) to capture areas of tissue with different stages of MmuPV1-induced cervicovaginal disease. To increase the likelihood of mice developing SCC to include in our analysis, we included groups of mice treated with exogenous estrogen (E2), which we have previously shown promotes viral persistence and exacerbates disease severity and cancer incidence in MmuPV1-infected mice (60, 64). In Experiment 5, tissues were collected from four groups of mice at the study endpoint (24 wpi): (i) mock-infected mice (Mock), (ii) mock-infected mice treated with estrogen (Mock + E2), (iii) MmuPV1-infected mice (MmuPV1), and (iv) MmuPV1-infected mice treated with estrogen (MmuPV1 + E2; Fig. 4A; Table 1). Only MmuPV1-infected mice that established persistent infections as determined by qPCR were included in this study. Regions of neoplastic disease were scored, marked by a trained pathologist, and collected using LCM. Precancerous lesions were considered either low-grade (CIN1/2) or high-grade (CIN2/3). In total, we captured areas of tissue representing no disease (*n* = 8), hyperplasia (*n* = 14), low-grade CIN (*n* = 7), high-grade CIN (*n* = 12), and SCC (*n* = 5) (Fig. 4F, left). From these collected lesions, DNA was isolated and 16S sequencing performed.

The average relative abundance of key bacterial genera within the vaginal communities for each of the four treatment groups is shown in Fig. 4B. We compared the microbial community composition in these samples based on several variables, including treatment group, sample location, estrogen treatment, MmuPV1 infection, viral load, and disease severity. Bray-Curtis beta diversity assessment revealed that neither the location within the female reproductive tract from which a sample was collected (ectocervix, cervicovaginal wall, vaginal wall, etc.) nor the estrus stage at the time of sample collection influences the microbiome (PERMANOVA; *P*-values > 0.1; Table 2). However, differences in microbial community composition trended toward significance between each of the four groups (Mock, Mock +E2, MmuPV1, MmuPV1 + E2), as well as between all untreated vs estrogen-treated mice and between all MmuPV1 vs Mock mice (all PERMANOVA *P*-values < 0.1; Fig. 4C; Table 2). PLS-DA analysis identified a higher abundance of *Streptococcus*, *Staphlyococcus*, and *Stenotrophomonas* in MmuPV1-infected local CVMs compared to mock-infected CVMs (Fig. 4D, Supplemental 5A). MASSLIN2 analysis identified reduced *Cutibacterium* in the local CVM of MmuPV1-infected mice (Fig. S5B).

We analyzed whether estrogen treatment significantly influenced microbial community composition in mice. In mock-infected mice, neither estrogen supplementation (*P*-value = 0.67) nor the stage of estrus (*P*-value = 0.34) at the time of collection had a significant effect on the local CVM (PERMANOVA; Table 2). In MmuPV1-infected mice, estrus stage was also not a significant factor (*P*-value = 0.89), although exogenous estrogen treatment significantly influenced the microbial community composition (*P*-value = 0.05). When we evaluated the association of estrogen treatment with vaginal microbial community composition in mock-infected and MmuPV1-infected groups separately, the community compositions were only significantly different in the MmuPV1-infected group (PERMANOVA; *P*-value = 0.05, Table 2). PLS-DA analysis identified key taxa associated with the local vaginal communities in MmuPV1 + E2 mice compared to untreated MmuPV1 mice (Fig. S5C), identifying higher abundance of *Streptococcus* and reduced *Micrococcus, Stenotrophomonas,* and *Pseudomonas*. However, none of these taxa were significantly different when assessed via MAASLIN2. Mock and Mock + E2 mice did not display significant differences in their community compositions. Conversely, microbial community compositions were significantly different between Mock + E2 and MmuPV1 +E2 mice (PERMANOVA; *P*-value < 0.05; Table 2) yet were not distinct between untreated Mock and MmuPV1 groups. We hypothesize that estrogen treatment, in part, through its positive effect on MmuPV1 viral copy number and disease severity (60, 64), likely augments infection-induced changes in local microbiome community composition.

We performed lavages prior to necropsy and used these to measure MmuPV1 viral copy number at the study endpoint using qPCR (Fig. 4E, left). Consistent with our prior observations (64), estrogen-treated MmuPV1-infected mice had higher viral loads than untreated MmuPV1-infected mice. When we compared the CVM of LCM samples collected from MmuPV1-infected mice with low versus high viral loads, the microbiome community compositions did not reach statistical significance (PERMANOVA; *P*-value = 0.08; Table 2), likely due to small sample size. PLS-DA analysis revealed that the CVM of mice with low viral load at the study endpoint was associated with higher relative abundance of *Micrococcus* and *Stenotrophomonas* (Fig. 4E, right; Fig. S5D).

Finally, we compared the LCM samples according to disease state. Overall, we did not detect significant differences in community composition between disease states present across all four groups of mice, between all infected mice, between untreated infected mice, or estrogen-treated infected mice (PERMANOVA; all *P*-values > 0.05; Table 2). Nevertheless, PLS-DA identified select microbial genera present within the local vaginal communities associated with low-grade dysplasia, high-grade dysplasia, and SCC (Fig. 4F, right; Fig. S5E). Comparing PLS-DA analyses between experiments, there was some degree of overlap between the globally- and locally-present microbial genera that distinguished disease states. In mice with low-grade disease (CIN1 and CIN2), our analyses identified higher relative abundance of *Pseudomonas* and *Corynebacterium* in the global cervicovaginal microbial communities (measured in lavage samples) in Experiment 4 (Fig. S3G). Similarly, *Pseudomonas* and *Corynebacterium* were also associated with low grade disease (roughly CIN2) at the local level in LCM samples (Experiment 5; Fig. 4F and Fig. S5E). In mice with more advanced disease, both global and local microbial communities were associated with *Staphylococcus* and *Stenotrophomonas* (i.e., the global CVM of mice with SCC in Experiment 4, Fig. S3G compared to the local CVM in CIN3 tissues in Experiment 5, Fig. S5E). We also identified genera at the local level within SCCs that we did not detect at the global level, including *Gemella* and *Anaerobacillus* (the local CVM in SCC tissues in Experiment 5, Fig. S5E compared to global CVM in mice with SCC in Experiment 4, Fig. S3G). Together, these results suggest that different stages of MmuPV1-induced neoplastic disease can influence both the global host CVM as well as the local microbial community structure within regions of disease. Future studies are warranted to determine the temporal nature of these changes and whether they are causally involved in disease development or simply inherent to different stages of neoplastic disease.

## DISCUSSION

The human microbiome is now well-recognized as a significant factor in several types of cancer (72), including cervical neoplastic disease and cancer (20, 73). Given the near complete association between high-risk HPV infections and cervical carcinogenesis (4), it is perhaps predictable that changes in the female cervicovaginal microbiome (CVM) correlated with cervical cancer are often inextricably linked with those induced by HPV infections and their resulting neoplasias (20, 22, 24, 25, 37). Our knowledge of this interplay is almost exclusively derived from human epidemiological studies. This makes preclinical models of papillomavirus pathogenesis and disease valuable tools that can be utilized to dissect underlying disease mechanisms. The discovery of a murine papillomavirus, MmuPV1, that infects common laboratory strains of mice has drastically enhanced our ability to model and interrogate HPV pathogenesis (44, 45). In this study, we sought to use our MmuPV1 infection model of cervicovaginal cancer (60) to define changes in the host CVM elicited by papillomavirus infection and disease. We measured significant variability in the naturally-occurring murine CVM among mice of similar age, genetic background, and housing conditions across multiple experiments (Fig. 1; Fig. S1). Nevertheless, MmuPV1 infection consistently influenced the host CVM composition in multiple experiments although the specific bacterial taxa altered in MmuPV1-infected mice varied (Fig. 2; Fig. S2). Significant changes in the CVM were observed across different temporal stages of MmuPV1 infection (Fig. 2; Fig. S2), as well as viral clearance versus persistence (Fig. 3; Fig. S3). We found that differences in MmuPV1 viral load, whether experimentally-induced (Fig. 3; Fig. S3) or naturally-acquired during the course of infection (Fig. S4), were associated with changes in the host CVM. Moreover, MmuPV1 infection resulted in a significant shift in microbial community composition in mice (Fig. 3). Finally, we discovered that MmuPV1-induced neoplastic disease severity influenced the composition of both the global microbiome (Fig. 3; Fig. S3 and 4) and intralesional and intratumoral local microbiome (Fig. 4; Fig. S5) in the female reproductive tract. To our knowledge, this is the first study to define the effects of papillomavirus infection and subsequent disease development on the CVM in an *in vivo* murine model.

When using preclinical animal model systems to study tumor virus-associated human diseases, one must consider their ability to accurately mimic aspects of both the host and pathogen (74). In the studies reported here, we are mindful of several key differences between the human and murine host CVM as well as HPV and MmuPV1 that are relevant to our interpretations. First, we observed significant variability across experiments in the natural CVM in unperturbed, uninfected *FVB/N* mice (Fig. 1; Fig. S1). This is consistent with other reports showing that the murine CVM exhibits significant instability and varies with genetic background, vendor, vivaria, and even within the same colony of mice (41, 42, 75–78). This variability underscores the challenging nature of studies such as ours and ultimately limited us to comparing trends across multiple experiments. A major difference between the human and mouse CVM worth noting is the dominance, or lack thereof, of *Lactobacillus* species in healthy murine CVMs. We only observed *Lactobacillus* as a core genus of the natural murine microbiome for mice in Experiment 2 (Fig. 1E). The healthy human CVM, however, is often dominated by *Lactobacillus* species (14, 15), a trait unique to humans and infrequently observed in rodents, primates, and other mammals (79). This discrepancy between murine and human CVMs may complicate model development and potentially limit the predictive power and ability to extrapolate experimental findings to human disease. As for the pathogen aspect of our model, there are differences between MmuPV1 and high-risk HPV viral genomes and the E6 and E7 viral oncoproteins (80, 81). However, both establish persistent viral infections and exhibit oncogenic potential in relevant anatomical sites (45, 55). For these reasons, the MmuPV1 cervicovaginal infection model is a relevant platform in which to study papillomaviruses and the CVM.

In experiments where we specifically monitored the natural CVM (Fig. 1A; Experiments 1 and 2), we identified 7 ‘core’ genera in the female reproductive tract of *FVB/N* mice that are members of the Actinomycetota, Bacillota, and Pseudomonadota phyla: *Acinetobacter*, *Corynebacterium*, *Micrococcus*, *Pseudomonas*, *Staphylococcus, Streptococcus*, and *Streptomyces* (Fig. 1E). This core CVM genera is largely distinct from those described for other common strains of laboratory mice, including *C57BL/6* (78), *BALB/C* (75), and *ICR* (77), and also differed from those described in other studies using *FVB/N* mice (41, 42). *Staphylococcus* and *Streptococcus* were routinely detected as major CVM taxa in multiple murine genetic backgrounds. Therefore, our study adds to the growing body of work describing the variable nature of the native CVM in laboratory mice. Of the core taxa we identified in *FVB/N* mice in our study, *Streptococcus*, *Staphylococcus*, and *Corynebacterium* are also commonly found in the human vaginal microbiome (14, 15, 82).

We observed several changes in the host CVM associated with MmuPV1 infection of the female reproductive tract. In multiple experiments, we identified differences between the microbial community structure of lavage samples taken prior to or following MmuPV1 infection within the same animal (Fig. 2 and Fig. S2; Table 2). We also determined that the CVM composition differs across each stage of MmuPV1 infection in multiple experiments (Fig. 2G; Fig. S2G and H; Fig. S2K and L). Together, these observations strongly suggest that there is a temporal element to the way in which MmuPV1 infection may influence the microbial community structure. Although we also observed significant changes in the host CVM between pre- and post-infection timepoints in mock-infected mice that are likely due to Depo treatment (Fig. S1; Table 2). Importantly, MmuPV1-infected mice displayed larger shifts in their CVM community composition over time compared to mock-infected mice (Fig. 3E). These changes are interesting considering our observations that disease severity increases over time in MmuPV1-infected mice (60). However, not all infected mice developed persistent infections (Fig. 3D) or severe neoplastic disease (Fig. 3I). It is possible that MmuPV1 infection, even if cleared or present at very low copy numbers, imparts long-lasting changes to the host CVM. This speculation is intriguing given evidence in humans that changes in the host microbiome can persist following viral clearance or disease regression either by natural means or surgical excision (38–40). Overall, these findings suggest that events that occur during later stages of papillomavirus infection, such as persistence, disease development and progression, and/or viral integration, may contribute to changes in the host CVM. MmuPV1 frequently integrates into the host genome in benign cutaneous papillomas (63) and it seems plausible that it also integrates in mucosal epithelia. Therefore, it would be interesting to use the MmuPV1 cervicovaginal model to explore the effect of viral integration on the host CVM.

Changes to the CVM observed upon MmuPV1 infection often correlated with key metrics of papillomavirus infection, such as viral load and viral persistence. MmuPV1 viral load was one of the more strongly correlated metrics influencing the host CVM in our study and was associated with changes across multiple experiments (Fig. 3D and F; Fig. S3A through D; Fig. S4A through B). Effects of viral load on the host CVM were observed in experiments where mice naturally developed either low or high-viral load infections over time and in mice with an experimentally-induced range of viral loads established through an inoculation dose curve. MmuPV1 viral clearance and persistence also influenced the composition of the host CVM (Fig. 3G; Fig. S3F). All mice developed persistent infections in Experiments 1 and 2, unfortunately, limiting our analysis of persistence versus clearance to only Experiments 3 and 4. While we observed differences between MmuPV1-infected mice that developed persistent infections compared to those that cleared infection, the CVM differences only reached significance in Experiment 4 (Table 2). Future studies are warranted to expand this area of research to more accurately determine the effect of MmuPV1 viral persistence on the host CVM. Likewise, studies should be performed to evaluate the role of inflammation and pro-inflammatory host CVM composition on MmuPV1 infection given recent findings that high levels of pro-inflammatory cytokines such as interleukin 1 beta (IL-1β) and interleukin-8 (IL-8) may be markers of inherent predisposition to HPV persistence and disease in women (20, 39, 40).

While MmuPV1 infection and key metrics clearly influenced the host CVM, the magnitude and effects on specific bacterial taxa varied widely, presumably due to the underlying variability in the natural microbiome observed in mice prior to infection (Fig. 1). However, there were specific taxa altered by MmuPV1 infection worth mentioning. Although *Lactobacillus* was not one of the core genera detected in the natural murine CVM in our study (Fig. 1), mice in Experiment 2 where *Lactobacillus* was present at baseline experienced a decline in *Lactobacillus* following MmuPV1 infection (Fig. 2D). We also observed an association between *Lactobacillus* presence and low viral loads and viral clearance in Experiment 4 (Fig. S3C and E). We did not measure the natural microbiome prior to infection in mice from Experiment 4 (Fig. 3A), so it is unclear if these mice had *Lactobacillus* present in high abundance prior to infection as with mice in Experiment 2. For this reason, it is similarly difficult to determine if fluctuations in the relative abundance of *Lactobacillus* can be causally correlated with viral load and clearance. Nonetheless, these observations are consistent with what often occurs to *Lactobacillus* species in HPV-infected women (22, 24, 37). *Cutibacterium* was also present in all MmuPV1-infected mice (Fig. 2I) but not in the core natural microbiome (Fig. 1E) although it was found as a core genus in post-Depo CVMs (Fig. 2E). This taxon was also identified as a top-driving taxa in analyses of MmuPV1 infection (Fig. 2F; Fig. S2E) and across different stages of MmuPV1 infection (Fig. 2H; Fig. S2G and K). In a retrospective study comparing women with high-grade dysplasia and SCC to a control group, *Cutibacterium* was identified as a previously unreported bacterial taxon present in cervical swabs (83). *In vitro*, a *Cutibacterium* species was found to alter various properties associated with epithelial barriers in HPV-immortalized keratinocytes (84). Considering these findings, it would be interesting to determine whether *Cutibacterium* is overrepresented in the CVM of mice that naturally acquire MmuPV1 in our sexual transmission model (59) or if it is simply a byproduct of Depo-provera treatment. The tractability of the MmuPV1 cervicovaginal preclinical model provides future opportunities to further explore the contribution of specific taxa to infection and disease outcomes.

There is evidence in humans that the microbiome differs between patients with different grades of dysplastic disease and cancer (21, 23, 25, 85). In this study, we evaluated the effect of different disease outcomes on both the global and local CVM in MmuPV1-infected mice. In several experiments (Experiments 1, 3, and 4), we measured the microbial composition of the host CVM present in lavages, which presumably capture bacteria from both diseased epithelia as well adjacent normal epithelia. While disease severity influenced the host CVM in all experiments to various degrees, the global CVM differences among disease states only reached significance in Experiment 4 where there was the greatest range in viral load and disease severity at the 24 week study endpoint (Table 2). Here, we identified several unique taxa specific to SCC that were not identified in any other global CVM throughout our studies: *Brochothrix*, *Rhodococcus*, and *Kocuria* ( Fig. S3G). The association between these taxa, if any, and HPV-related cancers is unclear, although *Rhodococcus* was upregulated in women with cervical dysplasia (86). It is worth noting that disease development and severity also correlated with changes to the host CVM in studies using HPV16 transgenic mice (41, 42), suggesting similarities may exist across models of HPV-induced disease.

The use of laser capture microdissection allowed us to survey the local, intratumoral CVM present in MmuPV1-induced SCCs. The bacterial taxa *Gemella* and *Anaerobacillus* were unique to the intratumoral CVM of SCCs (Fig. S5E), and these were not identified as driving taxa in the global CVM associated with SCC. Interestingly, Laniewski and colleagues identified *Gemella* as a novel bacteria associated with cervical HPV infection and disease stage in women (21). *Gemella* species were also identified in the microbiome of oral SCC tumors, but not adjacent normal tissue, from the same patient (87), although the HPV status of these patients was not noted. Our analyses suggest that there may be differences between the CVM measured in samples such as cervical swabs or lavages compared to discrete regions of epithelia containing specific grades of disease. In our studies, we did not collect lavages for the purpose of microbiome analysis (only for MmuPV1 viral load quantitation) in Experiment 5, so we were unable to directly compare the CVM composition of lavages to that found in LCM tissue. Likewise, we also did not compare global SCC CVM to local SCC CVM profiles given the extensive variability between different experiments. However, future studies should directly compare the global and local CVM from the same mice to more thoroughly explore whether there are informative differences. Furthermore, future longitudinal experiments can more closely address whether disease drives changes to the CVM or whether the CVM composition is a risk factor for future disease development. Data from human studies evaluating the CVM before and after disease regression, treatment, or surgical excision seem to support the latter theory (38, 39).

We have identified the female hormone estrogen as a host factor that exacerbates MmuPV1 viral load, viral persistence, and disease severity (60, 64). Estrogen-treated mice were included in Experiment 5 (Fig. 4A), thus allowing us to evaluate the effect of estrogen on the host CVM. In uninfected *FVB/N* mice, neither estrogen supplementation nor estrus stage significantly altered the host microbiome (Table 2), and this is consistent with observations from other studies (41, 42, 76, 78). However, we found estrogen supplementation was a significant contributor to changes in the CVM specifically in MmuPV1-infected mice (Table 2). Likewise, the host CVM in Mock + E2 mice versus MmuPV1 + E2 mice was also significantly different (Table 2). These findings suggest that the positive effects of estrogen on MmuPV1 viral load and disease severity we have observed and see in Experiment 5 (Fig. 4E and F) are most likely not due to direct estrogenic effects on the host CVM, but rather other estrogen-induced changes to other viral parameters or to the cervicovaginal microenvironment (88). Follow-up studies will seek to clarify the selective influence estrogen has on the host CVM in MmuPV1-infected, but not mock-infected, mice that our study revealed.

In conclusion, our study has characterized the effects of papillomavirus infection and pathogenesis on the host cervicovaginal microbiome in an *in vivo* murine model. We feel this study represents only the beginning of potential microbiome-related studies that can be pursued using the MmuPV1 cervicovaginal infection model. An obvious future direction is to evaluate how the microbiome influences key aspects of papillomavirus infection. Performing MmuPV1 infections of the female reproductive tract of germ-free mice or following manipulation of the host CVM by systemic antibiotic treatment (89) are both feasible experimental approaches for such studies. Studies involving colonization of mice with human bacteria can also be explored (76). The power of murine genetics also opens the door to evaluating the role of host genes and pathways in the interplay between the CVM and papillomavirus infection. The MmuPV1 cervicovaginal infection model, therefore, is a powerful and innovative tool that can greatly enhance our understanding of the interactions between the host microbiome and virus-induced cancers.

## MATERIALS AND METHODS

### Animals

All mice used in this study were 6- to 8-week-old wild-type *FVB/N* mice (Taconic Biosciences; Albany, NY) that were maintained using standard conditions. The female reproductive tract of all mice was colonized naturally from birth by the natural environment. All procedures were performed in a ventilated biosafety cabinet with laminar airflow and effort was taken to minimize influence by investigator and staff manipulation (cage changes, etc.). All animal experiments were performed in full compliance with standards outlined in the “Guide for the Care and Use of Laboratory Animals” by the Laboratory Animal Resources (LAR) as specified by the Animal Welfare Act (AWA) and Office of Laboratory Animal Welfare (OLAW) and approved by the Governing Board of the National Research Council (NRC). Mice were housed at McArdle Laboratory Animal Care Unit in strict accordance with guidelines approved by the Association for Assessment of Laboratory Animal Care (AALAC), at the University of Wisconsin Medical School. All protocols for animal work were approved by the University of Wisconsin Medical School Institutional Animal Care and Use Committee (IACUC, Protocol number: M005871).

### MmuPV1 infection and estrogen treatment

Mice were infected with MmuPV1 virus stock generated by isolating virions from papillomas that developed on infected *FoxN1^nu/nu^* mice as described previously (61). The female reproductive tract infection strategy was adapted from prior methods (90, 91) and has been described previously (60, 64). In mice that received exogenous estrogen, treatment was performed with a continuous-release pellet inserted subcutaneously as described previously (92, 93). Pellets were replaced every 2 months as needed.

### Vaginal lavage, DNA extraction, and MmuPV1 detection by qPCR

The method for collecting vaginal lavage DNA and detecting MmuPV1 DNA by quantitative PCR (qPCR) was modified from methods described previously (51, 55, 59, 64). DNA extraction was performed using spin columns (DNeasy Blood and Tissue kit; Qiagen #69506; Hilden, Germany). In each reaction, DNA was analyzed by real-time Sybr green PCR using primers specific to the murine 18sRNA gene and/or the MmuPV1 E2 gene. All qPCR was performed on an ABI 7900HT machine (Applied Biosystems).

### DNA extraction, library construction, and 16S sequencing

To process lavage samples for microbiome analysis, DNA extraction was performed as previously described with minor modifications (94). Briefly, samples were processed for yeast cell lysis (Epicentre, Lucigen; Middleton, WI) and resulting supernatants processed with PureLink Genomic DNA Mini Kit (Invitrogen; Waltham, MA) for DNA purification using the recommended protocol and eluted with PureLink Genomic Elution Buffer. DNA samples were sent for sequencing of the 16S rRNA gene V4 region at the University of Minnesota Genomics Center or sequencing of the 16S rRNA gene V3-V4 region at the University of Wisconsin Biotechnology Center. At both centers, amplicon libraries were constructed using a dual-indexing method and sequenced on a MiSeq with a 2 × 300 bp run format (Illumina, San Diego, CA). Swabs of mouse cage walls, the air in the mouse procedure area, and sterile PBS samples from the stock used for lavages and reagent-only negative controls were carried through the DNA extraction and sequencing process. An overview of the median raw sample reads and median reads after filtering out contaminants for each experiment is provided in Table S1.

### Sequence analysis

The QIIME2 environment was used to process DNA-based 16S rRNA gene amplicon data (95). Paired end reads were trimmed, quality filtered, and merged into amplicon sequence variants (ASVs) using DADA2. Taxonomy was assigned to ASVs using a naive Bayes classifier pre-trained on full length 16S rRNA gene 99% OTU reference sequences from the Greengenes database (version 13_8). Using the qiime2R package, data were imported into RStudio (version 1.4.1106) running R (version 4.2.1) for further analysis using the phyloseq package (96). ASVs identified within negative DNA extraction and sequencing controls were removed from all samples based on absolute read count and ASV distribution in mouse samples. The Bray-Curtis metric was utilized to assess beta-diversity between vaginal microbiome samples for each experiment. Type 3 (partial sum of squares) Univariate permutation ANOVAS with 9999-permutations were then used to determine whether sample microbial community beta diversity significantly clustered by intervention group(s), or infection outcomes (i.e., viral load, viral clearance or persistence at the endpoint, cervical dysplasia severity, etc.) (97).

### Prediction of microbial taxa associated with MmuPV1 infection outcomes

To predict the microbial genera associated with infection and infection outcomes (i.e., viral load, viral clearance or persistence, disease severity, etc.) microbiome data sets were integrated into supervised Partial Least Squares-Discriminant Analyses (PLS-DA) via the via MixOmics R-studio package (98). Vector plots were used to visualize the most influential microbial taxa that help distinguish outcome groups in each prediction model, for these plots the longer the vector (closer to 1), the greater the influence the taxa holds in potentially explaining the outcome. Microbiome Multivariable Association with Linear Models version 2 (MAASLIN2) (66) was also utilized to identify taxa significantly more or less abundant in mouse vaginal communities of various intervention and infection outcome groups. Since each experiment had its own baseline vaginal microbiome composition, PLS-DA and MAASLIN2 assessments were conducted for each experiment separately.

### Tissue procurement, processing, and histopathological analysis

For Experiments 1–4, reproductive tracts were harvested, fixed in 4% paraformaldehyde and paraffin-embedded. Every 10th H&E-stained serial section was evaluated by histopathological analysis and scored for worst disease by a trained pathologist (SMM*)* in the Department of Pathology and Laboratory Medicine (University of Wisconsin School of Medicine and Public Health). The scoring system is described in detail in Spurgeon *et al*. (60).

### Laser capture microdissection and DNA extraction

For Experiment 5, female reproductive tracts were collected as unfixed, frozen tissues embedded in light blue FSC 22 Frozen Section Media embedding compound (Catalog #3801481; Leica Biosystems; Wetzlar, Germany). Frozen tissues were sectioned and then evaluated by histopathological analysis. Regions to be extracted by laser capture microdissection (LCM) were located by a trained pathologist (SMM) in the Department of Pathology and Laboratory Medicine (University of Wisconsin School of Medicine and Public Health). Tissue regions of interest (ROIs) were captured using a PixCell II LCM system (Applied Biosystems/Arcturus) and added to a tube containing DNA/RNA Shield (Zymo Research; Irvine, CA) for microbiome nucleic acid analysis.

### Statistical analyses

Statistical analyses for microbiome data sets were conducted in R studio running R (version 4.2.1).

## Data Availability

Sequence reads for this project can be found under NCBI BioProject PRJNA1065017 at. Code for analysis and generation of figures can be found on GitHub at https://github.com/Kalan-Lab/Spurgeon_Townsend_etal_MmuPV1_VaginalMicrobiome.

## SUPPLEMENTAL MATERIAL

Figure S1 mbio.00933-24-s0001.tif Natural cervicovaginal microbial communities differ between experiments and are affected by Depo-Provera treatment.

Figure S2 mbio.00933-24-s0002.tif MmuPV1 infection shapes cervicovaginal microbial community composition.

Figure S3 mbio.00933-24-s0003.tif Initial MmuPV1 inoculation dose influences infection outcomes.

Figure S4 mbio.00933-24-s0004.tif Naturally occurring MmuPV1 viral load and neoplastic disease severity are associated with subtle changes in cervicovaginal microbiome composition.

Figure S5 mbio.00933-24-s0005.tif Influence of MmuPV1 infection and neoplastic disease severity on the local cervicovaginal microbiome.

Supplemental text mbio.00933-24-s0006.docx Supplemental Materials and Methods and figure and table legends.

Table S1 mbio.00933-24-s0007.docx Median raw sample reads and median reads after filtering out contaminants for each experiment.

ASM does not own the copyrights to Supplemental Material that may be linked to, or accessed through, an article. The authors have granted ASM a non-exclusive, world-wide license to publish the Supplemental Material files. Please contact the corresponding author directly for reuse.
