# Supplementary figures and images for: Key aspects of papillomavirus infection influence the host cervicovaginal microbiome in a preclinical murine papillomavirus (MmuPV1) infection model
Source: mBio. 2024 May 14;15(6):e00933-24. doi: 10.1128/mbio.00933-24 (PMC11237646; doi:10.1128/mbio.00933-24)

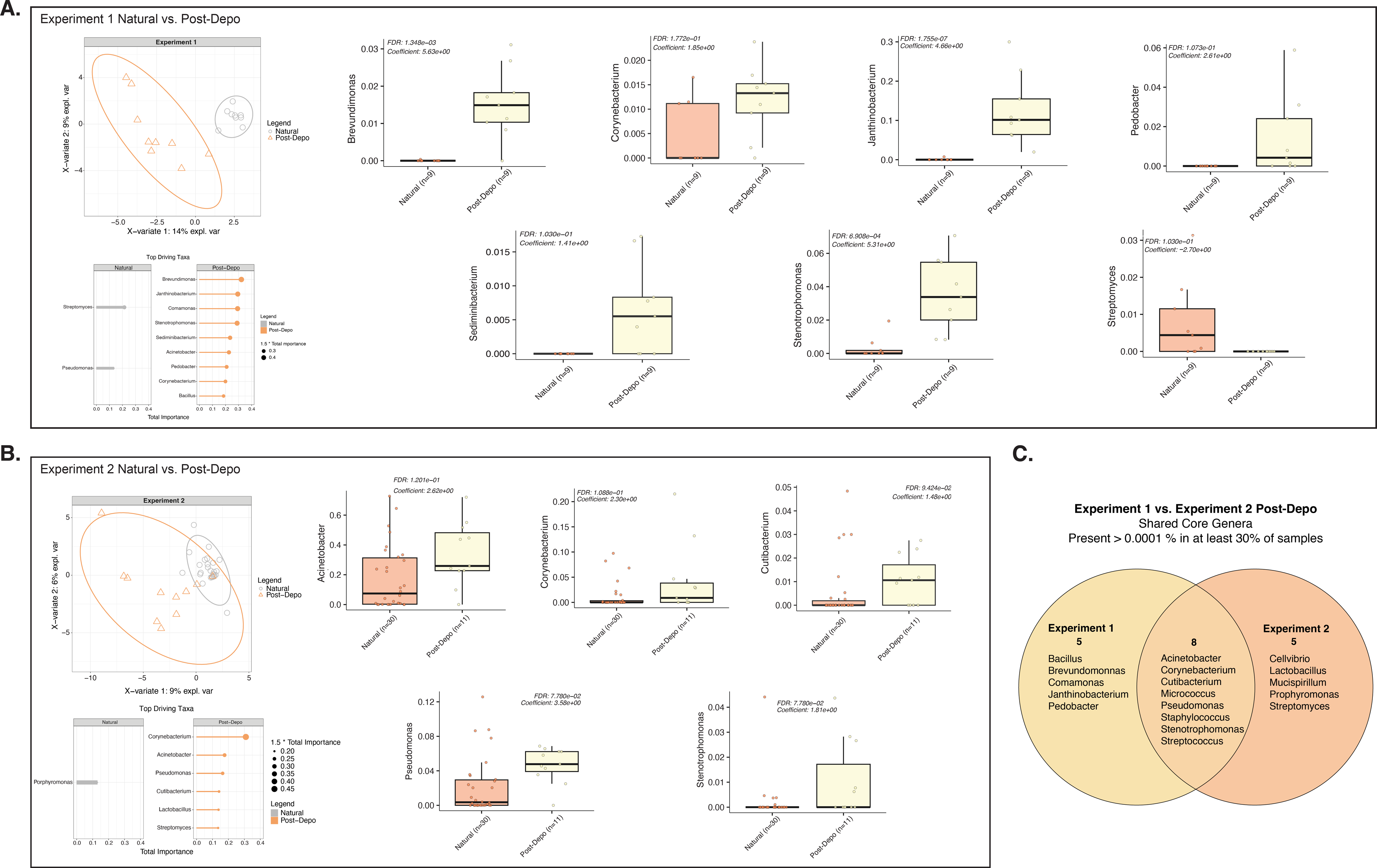

Supplement: Figure S1 — Natural cervicovaginal microbial communities differ between experiments and are affected by Depo-Provera treatment. [file mbio.00933-24-s0001.tif]

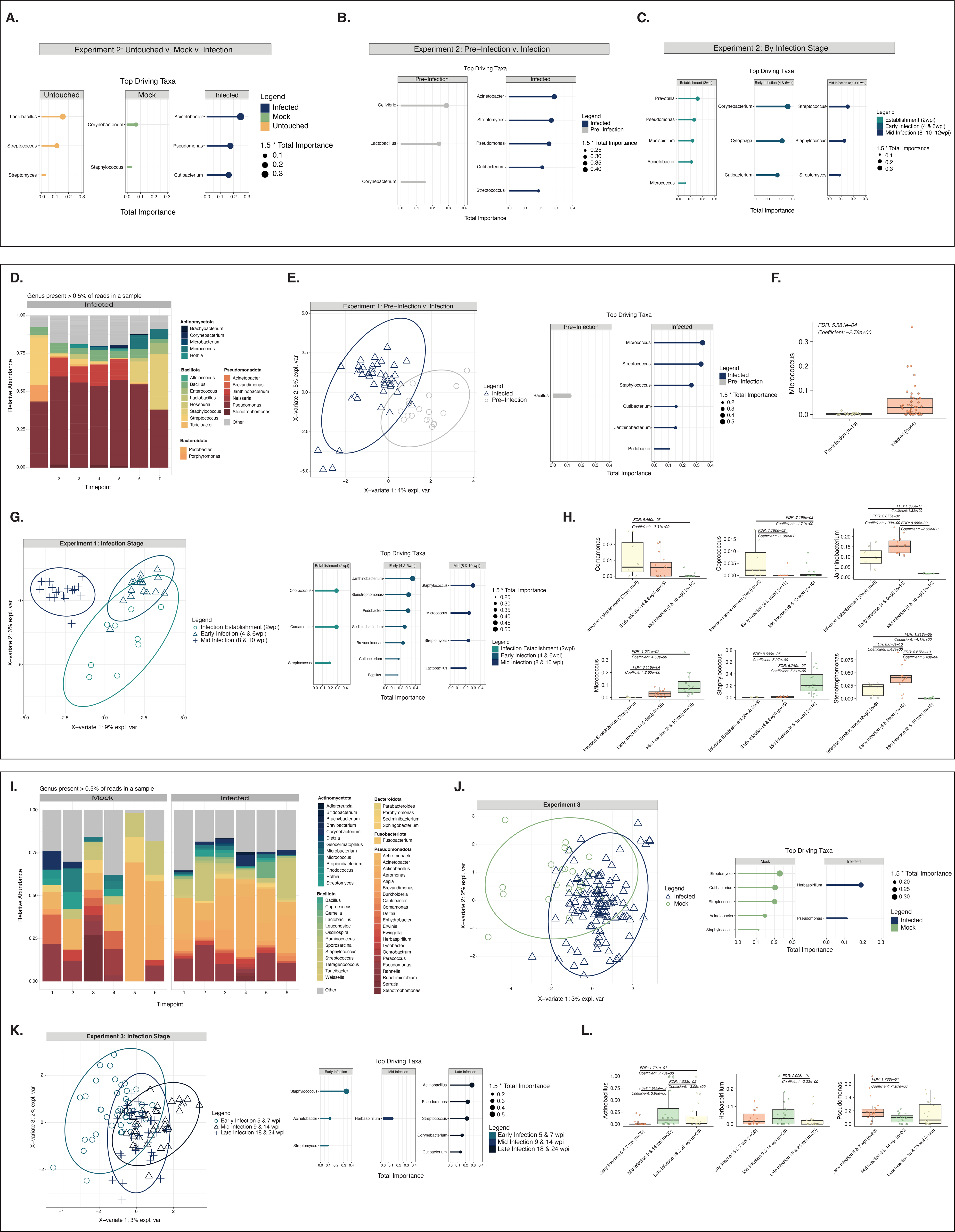

Supplement: Figure S2 — MmuPV1 infection shapes cervicovaginal microbial community composition. [file mbio.00933-24-s0002.tif]

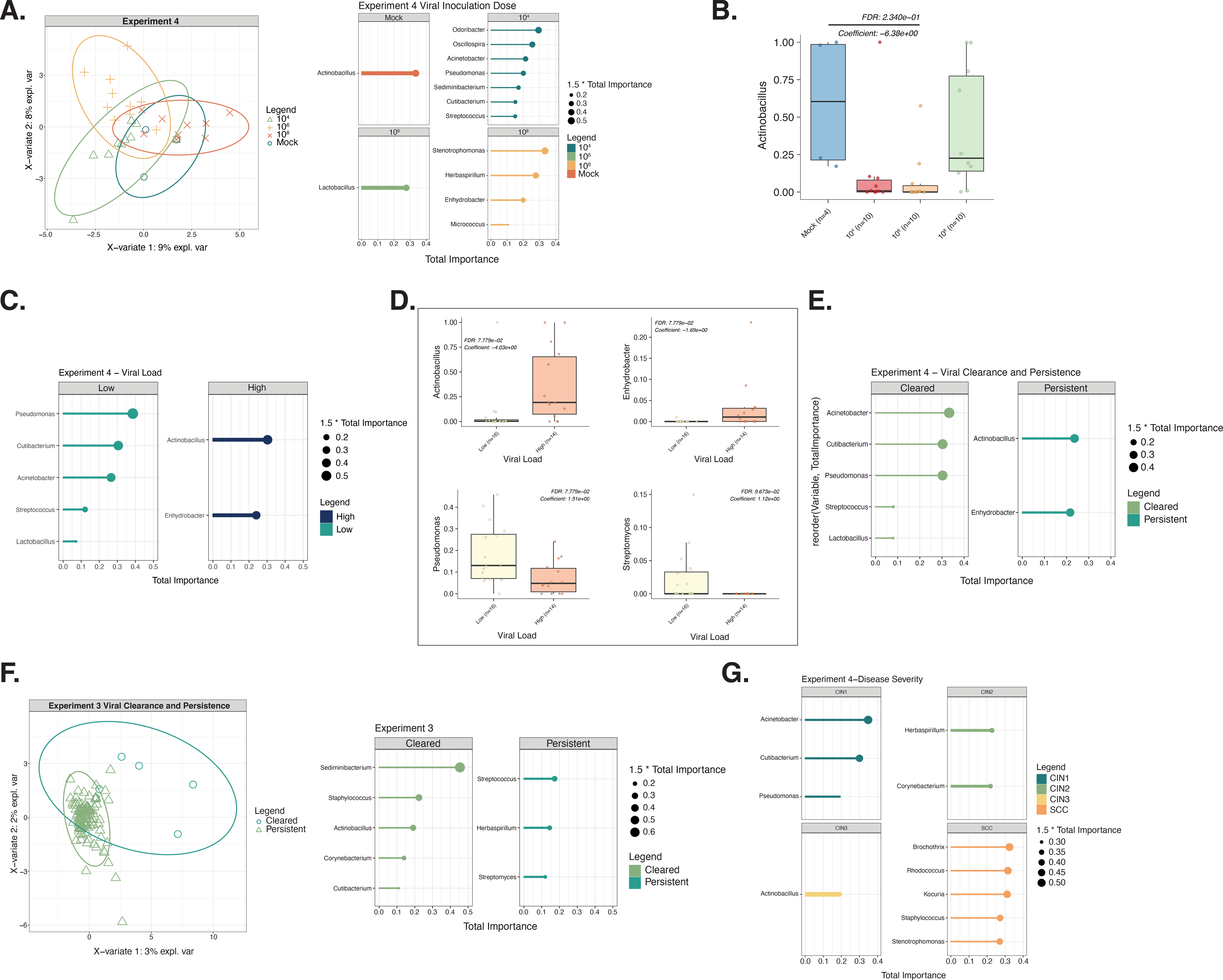

Supplement: Figure S3 — Initial MmuPV1 inoculation dose influences infection outcomes. [file mbio.00933-24-s0003.tif]

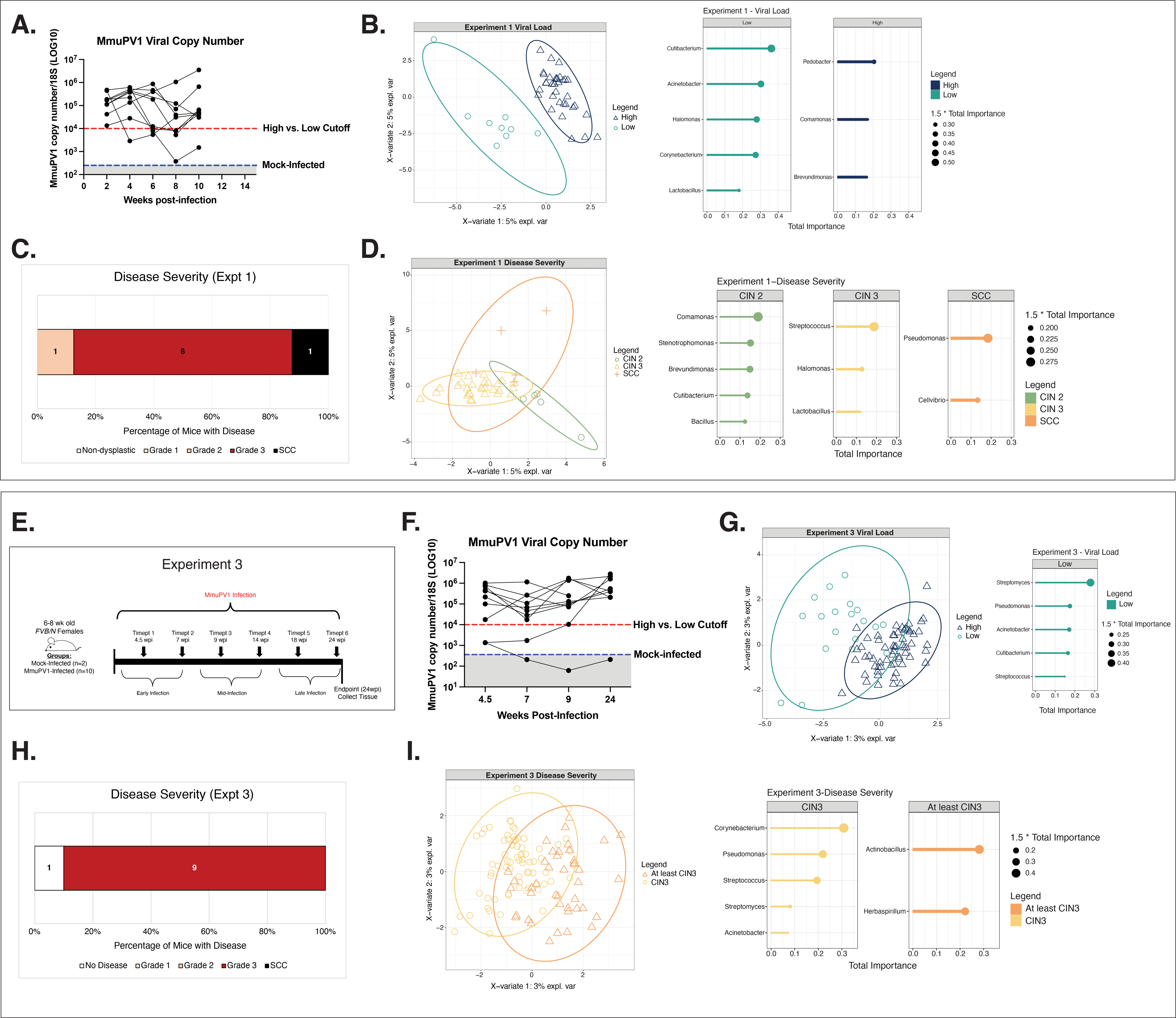

Supplement: Figure S4 — Naturally occurring MmuPV1 viral load and neoplastic disease severity are associated with subtle changes in cervicovaginal microbiome composition. [file mbio.00933-24-s0004.tif]

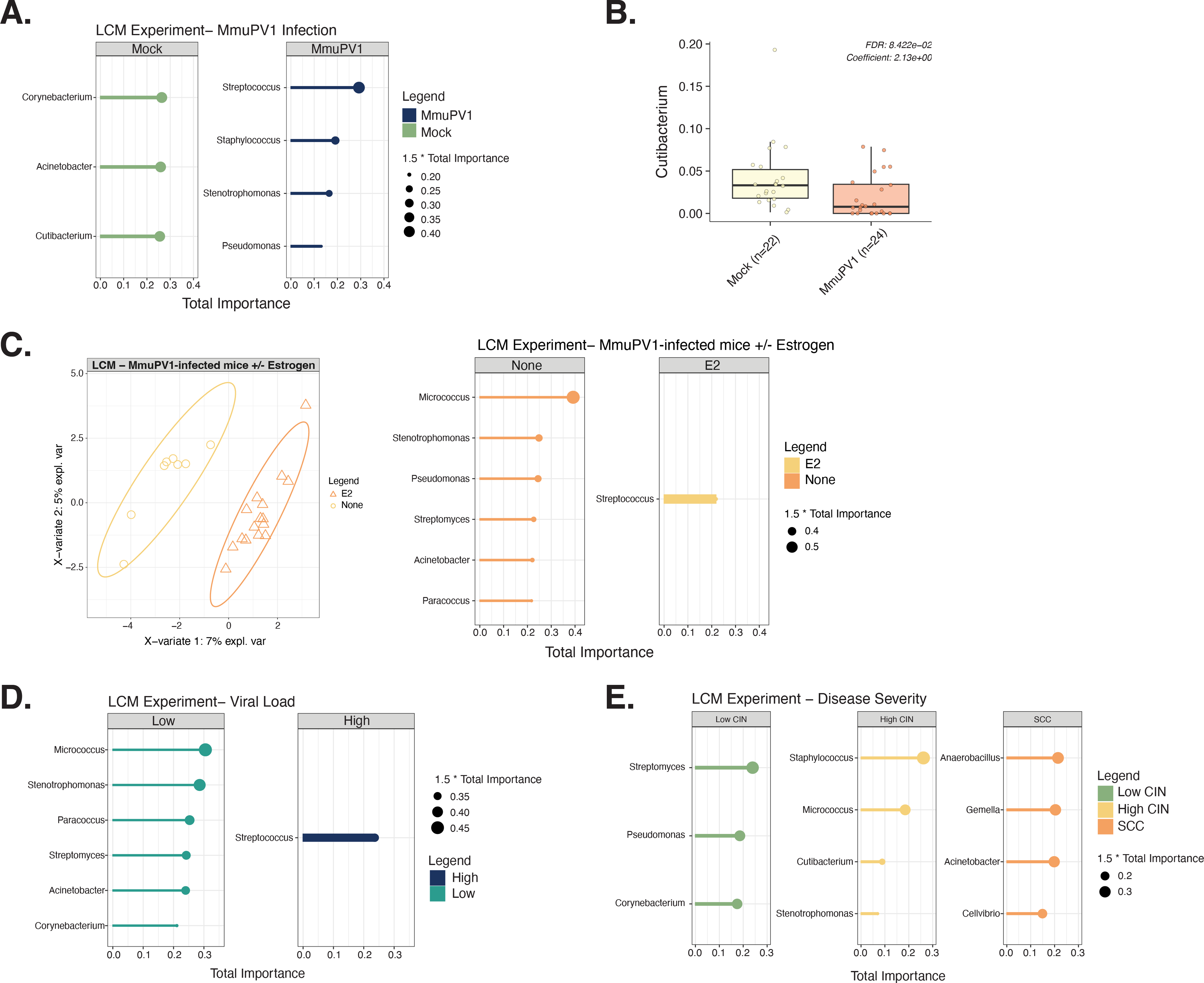

Supplement: Figure S5 — Influence of MmuPV1 infection and neoplastic disease severity on the local cervicovaginal microbiome. [file mbio.00933-24-s0005.tif]
