# Supplementary material for: Key aspects of papillomavirus infection influence the host cervicovaginal microbiome in a preclinical murine papillomavirus (MmuPV1) infection model
Source: mBio. 2024 May 14;15(6):e00933-24. doi: 10.1128/mbio.00933-24 (PMC11237646; doi:10.1128/mbio.00933-24)
Supplement: Supplemental text — Supplemental Materials and Methods and figure and table legends. [file mbio.00933-24-s0006.docx]

**SUPPLEMENTAL MATERIALS**

**SUPPLEMENTAL MATERIALS AND METHODS**

**Animals**

All mice used in this study were 6-8 week-old wild-type *FVB/N* mice (Taconic Biosciences; Albany, NY) that were maintained using standard conditions. All animals were randomly distributed and housed in plastic IVC (Individually Ventilated Cages) caging (Alternative Design Manufacturing & Supply, Inc; Siloam Spring, AR) containing Shepherd’s Cob + PLUS bedding containing corn cob bedding supplemented with ALPHA-twist nesting material (Shepherd Specialty Papers; Amherst, MA). Mice were housed in rooms with <60% humidity and a 72°F set point with alarm points outside a constant temperature range of 68-79°F. Housing rooms operate on 12-hour light/12-hour dark cycles. All mice were given ad libitum access to water and food (2019 Teklad Global 19% Protein Extruded Rodent Diet; Inotiv; Madison, WI). Except for animals used in Experiment 5 (LCM experiment), all mice were housed in the same room in the same vivarium and transferred to aseptic caging at the beginning of each experiment where they remained for the duration of each experiment. The female reproductive tract of all mice was colonized naturally from birth by the natural environment. All procedures were performed in a ventilated biosafety cabinet with laminar airflow and effort was taken to minimize influence by investigator and staff manipulation (cage changes, etc).

All animal experiments were performed in full compliance with standards outlined in the "Guide for the Care and Use of Laboratory Animals” by the Laboratory Animal Resources (LAR) as specified by the Animal Welfare Act (AWA) and Office of Laboratory Animal Welfare (OLAW) and approved by the Governing Board of the National Research Council (NRC). Mice were housed at McArdle Laboratory Animal Care Unit in strict accordance with guidelines approved by the Association for Assessment of Laboratory Animal Care (AALAC), at the University of Wisconsin Medical School. All protocols for animal work were approved by the University of Wisconsin Medical School Institutional Animal Care and Use Committee (IACUC, Protocol number: M005871).

**MmuPV1 infection and estrogen treatment**

Mice were infected with MmuPV1 virus stock generated by isolating virions from papillomas that developed on infected *FoxN1^nu/nu^* mice as described previously (61). The female reproductive tract infection strategy was adapted from methods described previously (90, 91). Briefly, mice were injected subcutaneously with 3 mg medroxyprogesterone acetate (Depo-Provera; Amphastar Pharmaceuticals, Rancho Cucamongo, CA) 4-7 days prior to MmuPV1 infection to induce diestrus. On the day of the infection, mice were pretreated vaginally with 50 µL VCF vaginal contraceptive gel containing 4% nonoxynol-9 (Apothecus Pharmaceutical Corporation, Ronkonkoma, NY; Catalog #247149) to induce chemical injury to the cervicovaginal epithelium (91). At 4 hours post-treatment with VCF, 10^8^ VGE (or 10^6^ and 10^4^ VGE in Experiment 4) MmuPV1 virions were suspended in 25 µL 4% carboxyl methylcellulose (CMC; Sigma, #C4888) and delivered intravaginally. Mock-infected animals were treated identically except no MmuPV1 virus was added to CMC. Untouched mice received no Depo-provera, VCF, or CMC treatments. Untouched, mock-infected, and MmuPV1-infected mice were housed in cages separate from other mice from different treatment groups. All treatments were performed while mice were anesthetized with 5% isoflurane.

In mice that received exogenous estrogen, treatment was performed as described previously (92, 93). Briefly, female mice were anesthetized with 5% isoflurane, and a continuous-release estrogen (E2) tablet (17β-estradiol; 0.05 mg/60 days; Innovative Research of America, Sarasota, FL) was inserted subcutaneously in the shoulder fat pads of the dorsal skin. For those mice receiving estrogen, treatment began 5 days following MmuPV1 infection. A new tablet was inserted every 2 months as needed.

**Vaginal lavage, DNA extraction, and MmuPV1 detection by qPCR**

The method for collecting vaginal lavage DNA and detecting MmuPV1 DNA by quantitative PCR (qPCR) was modified from methods described previously (51, 55, 59, 64). All cervicovaginal lavages were performed on mice in a biosafety cabinet with laminar airflow. While mice were anesthetized with 3% isoflurane, 25 μL of sterile PBS was introduced into the vaginal canal with a pipette tip and rinsed 4-5 times. This material was then pipetted into a sterile Eppendorf tube containing 25 μL of sterile PBS and mixed to achieve a total volume of 50 μL. From this mixture, half (25 μL) of the liquid lavage material was removed and placed into a separate sterile Eppendorf tube (designated as the “bacteria sample”) to be used for downstream microbiome analysis (described below). The remaining liquid in the original tube (designated as the “viral sample”) was used for MmuPV1 copy number/viral load analysis. To process lavage samples used in MmuPV1 copy number determination by qPCR, lavage samples were stored at -20°C until DNA extraction was performed using spin columns (DNeasy Blood and Tissue kit; Qiagen #69506; Hilden, Germany). Eluted DNA was quantified using a Nanodrop, and then dilutions of 5 ng/μL were prepared for each sample in deionized water. To perform quantitative PCR (qPCR) in Experiments 1-4, lavage DNA was analyzed in each reaction by time Sybr green PCR using primers to the MmuPV1 E2 gene and normalized using primers specific to a murine housekeeping gene (mouse 18sRNA gene) as described previously (55). The following primer sequences were used: MmuPV1_E2_1 (5’-GCCCGAAGACAACACCGCCACG-3’), MmuPV1_E2_2 (5’-CCTCCGCCTCGTCCCCAAATGG-3’), Mouse_18sRNA_1 (5’-CGCCGCTAGAGGTGAAATTC-3’), and Mouse_18sRNA_2 (5’-TTGGCAAATGCTTTCGCTC-3’). For Experiment 5, lavage DNA was quantified using a NanoDrop, and 5 ng was analyzed in each reaction by time Sybr green PCR using the same primers specific to the MmuPV1 E2 gene noted above (no normalization to 18S gene). Linearized MmuPV1 genome was used to generate a standard curve for each PCR plate. Sterile PBS samples from the stock used for lavages were included on each PCR plate to account for potential environmental contamination. All qPCR was performed on an ABI 7900HT machine (Applied Biosystems).

**DNA extraction, library construction, and 16S sequencing**

To process lavage samples for microbiome analysis, DNA extraction was performed as previously described with minor modifications (94). Briefly, 300 μl of yeast cell lysis solution (Epicentre MasterPure Yeast DNA Purification Kit), 0.3 μl of 31,500 U/μl ReadyLyse Lysozyme solution (Epicentre, Lucigen; Middleton, WI), 5 μl of 1 mg/ml mutanolysin (M9901, Sigma-Aldrich; St. Louis, MO), and 1.5 μl of 5 mg/ml lysostaphin (L7386, Sigma-Aldrich; St. Louis, MO) was added to 25 μl of mouse vaginal lavage samples or 100 µl Laser Capture Microdissection sample before incubation for one hour at 37°C with shaking. Samples were transferred to a 2 ml tube with 0.5 mm glass beads (Qiagen; Germantown, Maryland) and bead beat for 10 min at maximum speed on a Vortex-Genie 2 (Scientific Industries; Bohemia, NY), followed by a 30 min incubation at 65°C with shaking, 5 min incubation on ice. The sample was spun down at 10,000 rcf for 1 min and the supernatant was added to 150 μl of protein precipitation reagent (Epicentre, Lucigen; Middleton, WI) and vortexed for 10s. Samples were spun down at maximum speed (~21,000 rcf) and allowed to incubate at RT for 5 min. The resulting supernatant was mixed with 500 μl isopropanol and applied to a column from the PureLink Genomic DNA Mini Kit (Invitrogen; Waltham, MA) for DNA purification using the recommended protocol. To obtain as much DNA as possible, all samples were double eluted with 35 µl PureLink Genomic Elution Buffer. DNA extracted from vaginal lavages in experiments 1, 2, as well as the laser Capture microdissection samples were sent for sequencing of the 16S rRNA gene V4 region at the University of Minnesota Genomics Center. DNA extracted from vaginal lavages in experiments 3 and 4 were sent for sequencing of the 16S rRNA gene V3-V4 region at the University of Wisconsin Biotechnology Center. At both centers, amplicon libraries were constructed using a dual-indexing method and sequenced on a MiSeq with a 2x300 bp run format (Illumina, San Diego, CA). Sterile PBS samples from the stock used for lavages were included in each experiment to account for potential environmental contamination. Reagent-only negative controls were carried through the DNA extraction and sequencing process.

**Sequence analysis**

The QIIME2 environment was used to process DNA-based 16S rRNA gene amplicon data (95). Paired end reads were trimmed, quality filtered, and merged into amplicon sequence variants (ASVs) using DADA2. An overview of the median raw sample reads and median reads after filtering out contaminants for each experiment is provided in Supplemental Table 1. Taxonomy was assigned to ASVs using a naive Bayes classifier pre-trained on full length 16S rRNA gene 99% OTU reference sequences from the Greengenes database (version 13_8). Using the qiime2R package, data was imported into RStudio (version 1.4.1106) running R (version 4.2.1) for further analysis using the phyloseq package (96). Negative DNA extraction and sequencing controls were evaluated and ASVs in negative controls along with reads from mitochondria and chloroplast were removed from mouse microbiome samples. Samples were also filtered to remove ASVs with less than 10 reads in a sample or present in less than 5% of all vaginal microbiome samples from an experiment. Abundances were normalized proportionally to total reads per sample. Relative abundance plots were produced using the package ggplot2, where taxa below 0.5% relative abundance were pooled into an “Other” category. The Bray-Curtis metric was utilized to assess beta-diversity between vaginal microbiome samples for each experiment. Type 3 (partial sum of squares) Univariate permutation ANOVAS with 9999-permutations were then used to determine whether sample microbial community beta diversity significantly clustered by intervention group(s), or infection outcomes (ie viral load, viral clearance or persistence at the endpoint, cervical dysplasia severity, etc.) (97).

**Prediction of Microbial taxa associated with MmuPV1 infection outcomes.**

To predict the microbial genera associated with infection and infection outcomes (i.e. viral load, viral clearance or persistence, disease severity, etc.) microbiome datasets were integrated into supervised Partial Least Squares - Discriminant Analyses (PLS-DA) via the via MixOmics R-studio package (98). Vector plots were used to visualize the most influential microbial taxa that help distinguish outcome groups in each prediction model, for these plots the longer the vector (closer to 1), the greater the influence the taxa holds in potentially explaining the outcome. Microbiome Multivariable Association with Linear Models version 2 (MAASLIN2) (66) was also utilized to identify taxa significantly more or less abundant in mouse vaginal communities of various intervention and infection outcome groups. Since each experiment had its own baseline vaginal microbiome composition, PLS-DA and MAASLIN2 assessments were conducted for each experiment separately.

**Tissue procurement, Processing, and Histopathological Analysis**

For Experiments 1-4, reproductive tracts were harvested, fixed in 4% paraformaldehyde overnight and transferred to 70% ethanol before being embedded in paraffin. Serial sections (5 μm) were cut and every 10th section was stained with H&E. Tissue sections were evaluated by histopathological analysis and scored for worst disease by a trained pathologist in the Department of Pathology and Laboratory Medicine (University of Wisconsin School of Medicine and Public Health). The scoring system is described in detail in Spurgeon et al. (60). Images of H&E-stained cervical tumors and epithelia were captured using a Zeiss AxioImager M2 microscope and AxioVision software version 4.8.2 (Jena, Germany). Details regarding tissue collection and processing for laser capture microdissection are provided below.

**Laser capture microdissection and DNA extraction**

For Experiment 5, female reproductive tracts were collected as unfixed, frozen tissues embedded in light blue FSC 22 Frozen Section Media embedding compound (Catalog #3801481; Leica Biosystems; Wetzlar, Germany) and frozen on dry ice before storing at -80°C. Frozen tissues were sectioned at alternating thicknesses of 7 μm and 14 μm (7 μm sections used for H&E staining, 14 μm sections used for laser-capture microdissection) using a cryostat. Tissue sections were evaluated by histopathological analysis and lesions identified and regions to be extracted by LCM located by a trained pathologist in the Department of Pathology and Laboratory Medicine (University of Wisconsin School of Medicine and Public Health). The scoring system is described in detail in Spurgeon *et al*. (60). Determination of estrus stage was also determined by the pathologist’s histopathological analysis of tissue. Maps of reproductive tract tissue section marked with “regions of interest” (ROIs) were generated for use in subsequent laser capture microscopy (LCM).

Using the overview maps described above, ROIs from epithelium on microscope slide-mounted cervical tissue sections were extracted using LCM. Frozen sections were briefly stained in Mayer's Hematoxylin solution (Sigma-Aldrich), thoroughly dehydrated through successive increasingly concentrated ethanol washes, and finally washed with xylene (Fisher). Cells of interest were captured using a PixCell II LCM system (Applied Biosystems/Arcturus), which uses an infrared laser to melt cells to a thermoplastic film cap placed over the tissue section. The laser was pulsed at 100 mW for 9.6 ms with a focus spot size of 15 µm. Caps with attached cells were lifted off the slide and used to cap a 0.5 ml microfuge tube containing either TRIzol (Invitrogen) for RNA extraction or DNA/RNA Shield (Zymo Research) for microbiome nucleic acid analysis.

**Supplemental Figure 1: The natural cervicovaginal microbial communities differ between experiments and are affected by Depo-Provera treatment.** All analyses include data from Experiment 1 and Experiment 2. To predict key microbial genera that help distinguish cervicovaginal samples from before and after Depo-Provera treatment, microbial datasets from Experiments 1 and 2 were incorporated into supervised Partial Least Squares-Discriminant Analyses (PLS-DA). **A)** PLS-DA ordination (top) for Experiment 1 and corresponding vector plot (bottom) of key microbial genera that help distinguish the natural and post Depo-Provera groups in the supervised ordination. The longer the vector, the higher degree of influence the microbial taxa has in pulling a sample toward being in the natural or post Depo-Provera groups. The direction of the vector indicates that samples toward that side of the graph have higher relative abundance of the microbial genera. MAASLIN2 was utilized to confirm taxa significantly changed in mouse cervicovaginal communities either before or after Depo-Provera treatment in Experiment 1. These results are shown in the bar graphs on the right. For all MAASLIN2 plots, the y-axis indicates the relative abundance of the genera. For all significant comparisons, the False Discovery Rate (FDR) adjusted p-value with the Benjamini-Hochberg correction method along with the model coefficient value (effect size), indicating the degree of contrast between the selected category against the reference category (the “natural” microbiome, prior to any intervention), are shown. **B)** PLS-DA ordination for Experiment 2 (top) and corresponding vector plot (bottom) of key microbial genera that help distinguish the natural and post Depo-Provera groups in the supervised ordination. MAASLIN2 was utilized to confirm taxa significantly changed in mouse cervicovaginal communities either before or after Depo-Provera treatment in Experiment 2. These results are shown in the bar graphs on the right. **C)** Venn diagram of shared core taxa in mouse cervicovaginal microbial communities between Experiments 1 and 2 following Depo-Provera treatment. Diagram indicates taxa present in > 0.01% in at least 30% of the samples. Six of the eight core taxa (*Acinetobacter, Corynebacterium, Micrococcus, Pseudomonas, Staphylococcus,* and *Streptococcus*) are shared across mouse cervicovaginal communities both prior to intervention (natural; Figure 1E) and post-Depo-Provera in Experiments 1 and 2.

**Supplemental Figure 2. MmuPV1 infection shapes cervicovaginal microbial community composition.** All data shown in parts A-C are from Experiment 2. All data shown in parts D-H are from Experiment 1. All data shown in parts I-L are from Experiment 3. **A)** Supervised PLS-DA vector plot indicating key microbial genera that help distinguish microbial communities from untouched, mock-infected, and MmuPV1-infected groups of mice. This plot accompanies the supervised ordination shown in Figure 2C. **B)** Supervised PLS-DA vector plot indicating key microbial genera that help distinguish microbial communities from pre-infection or post-infection cervicovaginal microbiomes. This plot accompanies the supervised ordination shown in Figure 2E. **C)** Supervised PLS-DA vector plot indicating key microbial genera that help distinguish cervicovaginal microbial communities during infection establishment (2wpi), early infection (4-6wpi), and mid infection (8-10-12wpi). This plot accompanies the supervised ordination shown in Figure 2G. **D)** Relative abundance of microbial genera present in at least 0.5% of mouse cervicovaginal microbial communities from Experiment 1. Each bar represents the average relative abundance for mice within that group for a particular timepoint, ranging from pre-intervention (the “natural community”), 2 weeks post-Depo-Provera, as well as 2-10 weeks post-infection (wpi). **E)** Supervised PLS-DA ordination distinguishing samples from pre-infection or post-infection cervicovaginal microbiomes (left). Vector plot corresponding to the PLS-DA, indicating significant key microbial genera that distinguish samples from pre- and post-infected mice (right). **F)** Bacterial genera significantly more abundant in mice following infection identified via Microbiome Multivariable Association with Linear Models version 2 (MAASLIN2). For all MAASLIN2 plots, the y-axis indicates the relative abundance of the genera. For all significant comparisons, the False Discovery Rate (FDR) adjusted p-value with the Benjamini-Hochberg correction method along with the model coefficient value (effect size), indicating the degree of contrast between the selected category against the reference category (MmuPV1-infected mice), are shown. **G)** Supervised PLS-DA ordination distinguishing cervicovaginal microbial communities during infection establishment, early infection, and mid-infection (left). Corresponding vector plot, indicating key microbial genera that help distinguish each of the groups in the supervised ordination (right). **H)** Genera significantly more or less abundant in mouse cervicovaginal communities during infection establishment, early infection, or mid-infection via MAASLIN2. **I)** Relative abundance of microbial genera present in at least 0.5% of the cervicovaginal microbial communities from Experiment 3. Each bar represents the average relative abundance for mice within that group for a particular timepoint, ranging from 5-24 wpi or mock infection. **J)** Supervised PLS-DA ordination plot, distinguishing mock-infected v. MmuPV1-infected mice in Experiment 3 (left). Corresponding vector plot to ordination, indicating key microbial genera that distinguish cervicovaginal microbial communities from mock or infected mice (right). Of note, no significant differences in taxa relative abundance in mock-infected and the infected mice from Experiment 3 were observed via MAASLIN2. **K)** Supervised PLS-DA ordination distinguishing cervicovaginal microbial communities from infected mice during early infection (5-7 wpi), mid-infection (9-14 wpi) and late infection (18-25 wpi) (left). Corresponding vector plot to ordination, indicating key microbial genera that distinguish cervicovaginal microbial communities from infected mice during different infection stages (right). **L)** Genera significantly more or less abundant in infected mouse cervicovaginal microbial communities in early, mid, or late MmuPV1 infection. Differences were assessed via MAASLIN2.

**Supplemental Figure 3. Initial MmuPV1 inoculation dose influences infection outcomes. A)** Supervised PLS-DA distinguishing cervicovaginal microbial communities from mice in Experiment 4 that were mock-infected or inoculated with 10^4^, 10^6^, or 10^8^ viral genome equivalents (VGE) (left). Corresponding vector plot is shown on the right, indicating the key microbial genera that help distinguish the groups. The longer the vector the greater the influence the genera have in pulling the cervicovaginal microbiome sample toward the group’s cluster. **B)** Mice in the mock-infection and 10^8^ VGE infection groups of Experiment 4 have significantly greater relative abundance of *Actinobacillus* via MAASLIN2. **C)** A companion vector plot to the supervised PLS-DA ordination shown in Figure 3F that distinguishes cervicovaginal microbial communities from mice with low or high viral load at 8 and 20 wpi in Experiment 4. **D)** Microbial genera significantly more or less abundant in high or low viral load groups via MAASLIN 2 in Experiment 4. **E)** A companion vector plot to the supervised PLS-DA ordination shown in Figure 3G indicating the key microbial taxa that help distinguish the groups is shown on the bottom. **F)** Of n=10 total mice in Experiment 3, n=1 mouse cleared MmuPV1 viral infection (< 2.13x10^3^ VGE as measured by qPCR in mock-infected mice) and n=9 mice developed persistent infections at the 24 week post-infection (wpi) study endpoint. The plot on the left shows the supervised PLS-DA ordination of distinguishing cervicovaginal microbial communities from MmuPV1-infected mice with viral clearance or persistence at the endpoint. Cervicovaginal microbial communities from all timepoints were included in this assessment. The corresponding vector plot is shown on the right. We were unable to conduct similar analysis for the mice in Experiment 1, since all the mice had persistent viral infections at the study endpoint. **G)** A vector plot indicating key genera that help distinguish mouse cervicovaginal communities from mice with different stages of neoplastic disease. This is the corresponding vector plot to Figure 3I. No significant differences were observed between disease severity groups via MAASLIN2.

**Supplemental Figure 4.** **Across multiple experiments, naturally-occurring MmuPV1 viral load and neoplastic disease severity are associated with subtle changes in cervicovaginal microbiome composition.** All data shown in parts A-D are from Experiment 1 and data shown in parts E-I are from Experiment 3. **A)** Cervicovaginal lavages were collected, DNA extracted, and qPCR performed for the MmuPV1 E2 gene. Viral copy numbers were quantified at multiple timepoints across Experiment 1. Infected mice with viral copy numbers greater than 1x10^4^ copies were considered to have high viral load (red line). Values measured in mock-infected mice were considered as background levels (2.5x10^2^ copies, blue line). **B)** Supervised Partial Least Squares-Discriminant Analysis (PLS-DA) plot separating out mouse cervicovaginal microbial communities from mice with low versus high viral loads is shown on the left. Corresponding vector plots highlighting the key microbial genera that distinguish mice with low or high viral load are shown on the right. **C)** Overall disease severity scores resulting from histopathological analysis for Experiment 1. **D)** Supervised PLS-DA ordinations (left) for mice with different disease severity in Experiment 1. All post-infection timepoints for each mouse were included in the PLS-DA plots and points are colored by the cervical dysplasia score at the endpoint. A corresponding vector plot for Experiment 1 is shown on the right. Each plot displays the key microbial genera that help distinguish mouse cervicovaginal communities from mice with different stages of neoplastic disease including CIN2, CIN3, and SCC. No significant differences between cervical dysplasia groups were detected for Experiment 1 MAASLIN2. **E)** Schematic detailing the main hallmarks and timepoints sampled throughout Experiment 3. Arrows indicate times where cervicovaginal lavages were collected. Times labeled with “Timepoints” or “Timepts” indicate samples that were also screened by 16S sequencing. Numbers of mice per group are indicated. **F)** MmuPV1 viral copy numbers were quantified at multiple timepoints for Experiment 3. Infected mice with viral copy numbers greater than 1x10^4^ copies were considered to have high viral load (red line) and values measured in mock-infected mice represent background levels (3.62x10^2^, blue line). **G)** A corresponding supervised Partial Least Squares-Discriminant Analysis (PLS-DA) plot separating out mouse cervicovaginal microbial communities from mice with low or high viral loads is shown (left). A companion vector plot is shown (right) indicating the key microbial genera that distinguish cervicovaginal communities from mice with low or high MmuPV1 viral load for Experiment 3. The longer the vector, the higher degree of influence the microbial taxa has in pulling a sample toward being in the respective group. No significant differences were measured between low and high viral titer groups for Experiment 3 via MAASLIN2. **H)** Overall disease severity scores resulting from histopathological analysis of tissues collected from mice at the endpoint of Experiment 3. **I)** Supervised PLS-DA ordinations (left) and companion vector plot (right) for Experiment 3. All post-infection timepoints for each mouse were included in the PLS-DA plots and points are colored by the cervical dysplasia score at the endpoint. Each plot displays the key microbial genera that help distinguish mouse cervicovaginal communities from mice with different stages of neoplastic disease including CIN3 or at least CIN 3 (severe CIN3 but not SCC), which were the only stages of worst disease detected in Experiment 3.

**Supplemental Figure 5: Influence of MmuPV1 infection and neoplastic disease severity on the local cervicovaginal microbiome. A)** Companion vector plot to Figure 4D indicating the key microbial genera that help distinguish local cervicovaginal microbial communities between mock-infected and MmuPV1-infected mice. **B)** Genera significantly more or less abundant in mock-infected versus MmuPV1-infected mice by MAASLIN2 analysis. **C)** Supervised PLS-DA ordination distinguishing local cervicovaginal microbial communities from MmuPV1-infected mice treated with estrogen versus those without estrogen treatment in Experiment 5 (left). All MmuPV1-infected mice were included in this analysis. Companion vector plot indicating the key microbial genera that help distinguish local cervicovaginal microbial communities is shown on the right. **D)** Companion vector plot to Figure 4E**,** indicating key genera that help distinguish mice with low or high viral load. Note no significant differences in taxa relative abundance were observed between mice in the high and low viral load groups via MAASLIN2. **E)** Vector plot displaying key taxa that help differentiate mouse cervicovaginal communities from mice with different levels of neoplastic disease severity. This plot is the companion to Figure 4F. Note that no significant differences in taxa relative abundance were observed between disease severity groups via MAASLIN2.

**Supplemental Table 1.** Table displays median raw sample reads and median reads after filtering out contaminants for each experiment.
