# Supplementary material for: Key aspects of papillomavirus infection influence the host cervicovaginal microbiome in a preclinical murine papillomavirus (MmuPV1) infection model
Source: mBio. 2024 May 14;15(6):e00933-24. doi: 10.1128/mbio.00933-24 (PMC11237646; doi:10.1128/mbio.00933-24)
Supplement: Table S1 — Median raw sample reads and median reads after filtering out contaminants for each experiment. [file mbio.00933-24-s0007.docx]

**Supplemental Table 1.** Table displays median raw sample reads and median reads after filtering out contaminants for each experiment.

| **Experiment** | **16s Region Sequenced** | **Raw Sequence Reads** | **Reads After Filtering** |
| --- | --- | --- | --- |
| Experiment 1 | V4 | 27627 | 11156 |
| Experiment 2 | V4 | 16962 | 1177 |
| Experiment 3 | V3-V4 | 11340 | 2373 |
| Experiment 4 | V3-V4 | 7921 | 1833 |
| Experiment 5 (Laser Capture Microdissection) | V4 | 8499 | 3805 |
